# PVT1/miR-136/Sox2/UPF1 axis regulates the malignant phenotypes of endometrial cancer stem cells

**DOI:** 10.1038/s41419-023-05651-0

**Published:** 2023-03-03

**Authors:** Qing Li, Fanfei Kong, Rong Cong, Jian Ma, Cuicui Wang, Xiaoxin Ma

**Affiliations:** Department of Obstetrics and Gynecology, Shengjing Hospital of China Medical University, 39 Huaxiang Road, Tiexi District, Shenyang City, Liaoning Province 110022 China

**Keywords:** Endometrial cancer, Cancer stem cells

## Abstract

Tumor stem cells (TSCs) are thought to contribute to the progression and maintenance of cancer. Previous studies have suggested that plasmacytoma variant translocation 1 (PVT1) has a tumor-promoting effect on endometrial cancer; however, its mechanism of action in endometrial cancer stem cells (ECSCs) is unknown. Here, we found that PVT1 was highly expressed in endometrial cancers and ECSCs, correlated with poor patient prognosis, promoted the malignant behavior and the stemness of endometrial cancer cells (ECCs) and ECSCs. In contrast, miR-136, which was lowly expressed in endometrial cancer and ECSCs, had the opposite effect, and knockdown miR-136 inhibited the anticancer effects of down-regulated PVT1. PVT1 affected miR-136 specifically binding the 3’ UTR region of Sox2 by competitively “sponging” miR-136, thus positively saving Sox2. Sox2 promoted the malignant behavior and the stemness of ECCs and ECSCs, and overexpression Sox2 inhibited the anticancer effects of up-regulated miR-136. Sox2 can act as a transcription factor to positively regulate Up-frameshift protein 1 (UPF1) expression, thereby exerting a tumor-promoting effect on endometrial cancer. In nude mice, simultaneously downregulating PVT1 and upregulating miR-136 exerted the strongest antitumor effect. We demonstrate that the PVT1/miR-136/Sox2/UPF1 axis plays an important role in the progression and maintenance of endometrial cancer. The results suggest a novel target for endometrial cancer therapies.

## Background

Endometrial cancer is one of the three major malignant tumors of the female reproductive system, accounting for 20–30% of gynecological malignant tumors [1]. Due to the rapid economic growth in recent years, changes in people’s diets and living habits, and inappropriate hormone therapy, the incidence of endometrial cancer has gradually increased, especially in the younger population [2, 3]. At present, the treatment of endometrial cancer is mainly based on surgery. For patients with advanced stage or endometrial cancer recurrence, only radiotherapy, chemotherapy, progesterone, or targeted drugs can be used, but the curative effects are unsustainable and low. It is therefore important to elucidate the mechanisms of occurrence and development of endometrial cancer and its proposed targeted treatment methods.

The characteristics of tumor cell growth, metastasis, and recurrence are similar to the basic characteristics of stem cells. Therefore, some scholars have put forth the theory of tumor stem cells (TSCs). The American Association for Cancer Research defines TSCs as cells in a tumor that have the ability to self-renew and generate heterogeneous tumor cells. TSCs are extremely rare, and their tumorigenic ability, which is the basis for the occurrence, development, and maintenance of tumors, is significantly greater than that of ordinary tumor cells [4]. TSCs have provided a breakthrough in the basic and clinical theory of tumors, which will have a profound impact on the understanding of the occurrence, development, clinical diagnosis, and treatment of tumors. Researchers have extracted endometrial cancer stem cells (ECSCs) from the estrogen-dependent well-differentiated Ishikawa cell line [5].

Long non-coding RNAs (lncRNAs) are involved in the regulation of normal human physiology and the pathological processes of tumors, inflammation, and other diseases. Studies have found that a variety of lncRNAs are closely related to the factors involved in the occurrence and development of endometrial cancer, including plasmacytoma variant translocation 1 (PVT1). PVT1 is a tumor-specific gene located on human chromosome 8q24.21. It plays an important role in clear cell renal cell cancer [6], breast cancer [7], gastric cancer [8], and other tumors, and can promote the malignant biological behavior of endometrial cancer [9, 10]. However, the mechanism of action of PVT1 in ECSCs remains unclear.

The 3’ UTRs of some lncRNAs contain microRNA (miRNA) response elements (MREs) [11], which can adsorb certain miRNAs and regulate the expression of their target genes. The microRNA.org bioinformatics system analysis found that the MRE of PVT1 has a conserved binding site with miR-136. Some studies suggest that miR-136 plays a role in the progression, diagnosis, and treatment of malignant tumors, such as gastric cancer [12], bladder cancer [13], liver cancer [14], and lung cancer [15, 16], but few studies have investigated endometrial cancer.

Sex-determining region of Y chromosome (SRY)-related high-mobility-group box 2 (Sox2) is located at 3q26.33. Sox2 can cooperate with the transcription factors Oct4, Klf4, and c-Myc to recode differentiated somatic cells into induced pluripotent stem cells. It plays an important role in the regulation of embryonic development and maintenance of the differentiation ability of stem cells [17, 18]. Sox2 is also a cancer stem cell marker currently recognized by researchers [19]. The elevation of Sox2 is positively correlated with poor prognosis in endometrial cancer [20]. TargetScan, however, predicted that miR-136 could base-pair with the 3’-UTR region of Sox2 and regulate the expression of Sox2 at the post-transcriptional level.

We predicted that Sox2 could act as a transcription factor for Up-frameshift protein 1 (UPF1) by binding to the CCAAT sequence upstream of the UPF1 transcriptional start point. UPF1 is involved in the nonsense-mediated RNA decay (NMD) pathway [21] and plays an important role in DNA replication [22]. The overexpression of UPF1 in neuronal stem cells favors cell proliferation and maintains a stem cell-like state [23]. Moreover, UPF1 expression is elevated in rectal cancer and maintains rectal cancer stem cell stemness [24]. In studies of endometrial cancer, UPF1 expression has been found to be elevated and promotes the stemness of ECSCs [25].

We aimed to investigate the mechanism by which PVT1 regulates the expression of Sox2 and UPF1 by targeting miR-136, thereby affecting the malignant biological behavior and cell stemness of endometrial cancer cells (ECCs) and ECSCs. We further provided new ideas for understanding the pathogenesis and treatment of endometrial cancer.

## Materials and methods

### Human tissue specimens

A total of 55 endometrial cancer tissues and 30 normal endometrial tissues were obtained from patients who underwent hysterectomies in the Shengjing Hospital of China Medical University from 2013 to 2017. The patients’ age, International Federation of Obstetrics and Gynecology (FIGO) stage, histological grade, invasion depth, lymphovascular space invasion (LVSI), lymphatic metastasis, and distal metastasis data were collected from the electronic medical record system, and the survival data were obtained by telephone follow-up. All patients signed the informed consent. Specimens were evaluated by two pathologists. The patients recruited in this study did not receive chemotherapy or radiotherapy before surgery. The collection of tissue samples and patients’ information was reviewed and approved by the Scientific Research and New Technology Ethical Committee of the Shengjing Hospital of China Medical University (No. 2018PS251K).

### Cell lines and cell culture

The human endometrial cancer cell line Ishikawa was provided by the Department of Pathophysiology at Peking University (Beijing, China) in RPMI1640 medium (Gibco, Carlsbad, CA, USA) supplemented with 10% fetal bovine serum (FBS; Gibco) and 1% penicillin-streptomycin (Invitrogen, Carlsbad, CA, USA). The human embryonic kidney (HEK) 293T cells were purchased from the Shanghai Institute of Cell Biology at the Chinese Academy of Sciences (Shanghai, China). The HEK 293T cells were cultured in high glucose DMEM (Gibco). Non-stem cells and ECSCs were isolated from the Ishikawa cell line. Non-stem cells were cultured using the same method as the Ishikawa cells. ECSCs were grown in serum-free medium (SFM) containing DMEM/F12 (1:1) (Gibco), 2% B27 supplements (Gibco), 20 ng/ml epidermal growth factor (EGF), basic fibroblast growth factor (bFGF) (Peprotech, Rocky Hill, USA), 1% InsulinTransferrin-Selenium, 5% bovine serum albumin (BSA) (Roche, Basel, Switzerland), HEPES (Amresco, Solon, USA), and 1% penicillin-streptomycin (Invitrogen). The ECSCs were then suspended in a 6-well low attachment surface well plate (Corning, NY, USA) [5]. All cells were cultured in a humidified incubator at 37 °C with 5% carbon dioxide.

### Stem cells sorting

Ishikawa cells were suspended cultured in stem cell culture medium for 24 h, the suspended cells were digested into single cells with trypsin-EDTA (0.25%, Solarbio, Beijing, China). The single cell suspension (100 µL buffer/10^7^ cells) was incubated with PE-CD133, PE-Cy7-CD44, and isotype control antibodies (BD, Franklin Lakes, NJ, USA) at 4 °C in the dark for 30 min, and the cells were sorted by flow cytometry (FACSARia, BD, USA). The CD44+/CD133+ cells obtained through sorting were considered as ECSCs, and other cells were non-stem cells.

### Reverse transcription and quantitative real-time PCR (qRT-PCR)

Total RNA was extracted from tissues and cells using the Trizol reagent (Takara, Dalian, China). The cDNA of lncRNA or mRNA was synthesized with the PrimeScript RT Reagent Kit with gDNA Eraser (Takara, Dalian, China). The cDNA of miR-136 was synthesized with the Mir-X miRNA First Strand Synthesis Kit (Takara, Dalian, China). Real-time PCR was performed on the ABI Prism 7500 Fast Real-Time PCR System (Applied Biosystems, StepOnePlus, USA) using the SYBR Premix Ex Taq Kit (Takara, Dalian, China). U6-snRNA was used as the internal control of miR-136, and glyceraldehyde phosphate dehydrogenase (GAPDH) was used as the internal control of lncRNA or mRNA. The relative expression of RNAs was calculated using the comparative 2^−ΔΔCt^ method. All primer sequences are listed in Table S1.

### Western blot

Total proteins were isolated from cultured cells and tissues, separated on 8%, 10%, and 12% gels by sodium dodecyl sulfate polyacrylamide gel electrophoresis, and transferred onto polyvinylidene difluoride (PVDF) membranes. The PVDF membranes were incubated with polyclonal mouse anti-Sox2 antibody (1:5000; Proteintech), monoclonal rabbit anti-UPF1 antibody (1:50,000; Abcam), monoclonal rabbit anti-CD133 antibody, monoclonal rabbit anti-CD44 antibody, monoclonal rabbit anti-Oct4 antibody, monoclonal rabbit anti-Nanog antibody (1:1000; CST), monoclonal mouse anti-GAPDH antibody, and monoclonal mouse anti-β-Actin antibody (1:10,000; Proteintech) overnight at 4 °C. The PVDF membranes were then washed with TBST and incubated with horseradish peroxidase-conjugated anti-rabbit or anti-mouse secondary antibody (1:10,000; Proteintech) for 1 h at room temperature. Protein bands were visualized with enhanced chemiluminescence (Thermo Scientific, Carlsbad, CA, USA) in Image Lab software (Bio-Rad, CA, USA). The protein bands were normalized to GAPDH or β-Actin, and the relative integrated density values (IDVs) were calculated by the ImageJ software.

### Transfection of cells

Overexpression plasmids (pcDNA3.1-PVT1, pEX4-Sox2), mimic-miR-136, knockdown plasmids (sh-Sox2), inhibitor-miR-136, and their respective negative controls (NC) were purchased from GenePharma (Shanghai, China). They were then transfected into cells using a transfection reagent (jetPRIME, NY, USA) according to the manufacturer’s instructions. The lentivirus low-expression plasmids harboring PVT1 (LV-PVT1-RNAi 47488-1) and NC lentivirus (hU6-MCS Ubiquitin EGFP-IRES-puromycin) were purchased from GenePharma (Shanghai, China) and were transfected at a multiplicity of infection (MOI) of 100 [10]. The efficacy of overexpression and silencing was detected by qRT-PCR. The sequences of the lentivirus, plasmids, mimic, and inhibitor are listed in Table S2.

### Dual-luciferase reporter assay

Based on the predicted binding site of PVT1 and miR-136 and the binding site of Sox2 and miR-136 by the bioinformatics system, we commissioned GenePharma (Shanghai, China) to design and synthesize wild-type doublets containing the 3’-UTR region. The dual-luciferase vectors were PVT1-wild-type (PVT1-WT) and Sox2-wild-type (Sox2-WT), and their corresponding wild-type dual-luciferase vectors were PVT1-mutated-type (PVT1-Mut) and Sox2-mutated-type (Sox2-Mut). WT or Mut were co-transfected with miR-136 mimics or NC into HEK 293T cells. The relative luciferase activity was detected by the Dual-Luciferase Reporter Assay System (Promega, WI, USA) 48 h later. Based on the predicted binding sites, dual-luciferase vectors (GenePharma, Shanghai, China) containing the UPF1 promoter region were designed, synthesized, and co-transfected with pEX4-Sox2 or pEX4-NC into HEK 293T cells. The relative luciferase activity was detected after 48 h.

### Fluorescence in situ hybridization (FISH)

The subcellular localization of PVT1 and miR-136 was identified by FISH. Cell clipping sheets were made, cells were fixed with 4% paraformaldehyde, permeated with 0.1% Triton X-100, and blocked with BSA. Hybridized the cells with the probe (Genepharma, Shanghai, China) at 37 °C in dark for 14 h. Then the cells were washed by SSC solution, and the nuclei were stained with DAPI for 15 min without light. Finally, the cells were photographed under the fluorescence microscope.

### Cell proliferation assay

Cells were cultured in 96-well plates at a density of 3000 cells per well. The Cell Counting Kit-8 (CCK8; Dojindo Molecular Technologies, Kumamoto, Japan) was added 24 h after transfection. After an additional 4 h of incubation, the absorbance was measured at 450 nm using the SpectraMax M5 microplate reader (Molecular Devices, USA). Then added CCK8 every 24 h and measured the absorbance at 450 nm until 96 h after transfection.

Cells were treated with the 5-ethynyl-2’-deoxyuridine (EdU) kit (RiboBio, Guangzhou, China) according to the manufacturer’s protocol. They were then photographed under a fluorescence microscope to calculate the proportion of proliferating cells.

### Transwell migration and invasion assay

Cell migration and invasion abilities were detected using 24-well transwell chambers (8 µM pore size; Corning, NY, USA). Complete medium (500 mL) was added to each well followed by the transwell chambers. Serum-free medium (200 mL) containing 10^5^ single cells was added evenly to the upper chambers and incubated in a humidified incubator at 37 °C with 5% carbon dioxide. After 20 h, the transwell chambers were removed and washed. The cells were fixed, stained, and photographed under a microscope to count the number of migrated cells. For the invasion assays, Matrigel solution (BD Biosciences, Franklin Lakes, NJ, USA) was pre-applied to polycarbonate membranes in the transwell upper chambers. The remainder of the protocol was the same as that of the migration assays.

### Cell apoptosis assay

The cells were digested into a single-cell suspension. Annexin V-phycoerythrin/7-amino-actinomycin D (PE/7AAD; KeyGEN BioTECH, Nanjing, China) was added according to the manufacturer’s instructions. The cells were incubated at room temperature for 15 min in the dark. They were then analyzed by flow cytometry (BD FACSCalibur; BD Biosciences, NJ, USA).

### Cell cycle analysis

The cells were digested into a single-cell suspension, washed with 70% alcohol, then fixed for 4 h to overnight at 4 °C. Afterward, appropriate amounts of RNase A (KeyGEN BioTECH, Nanjing, China) and propidium (PI) (KeyGEN BioTECH, Nanjing, China) were added to the cell suspension. The cells were incubated at 37 °C for 30 min in the dark. Flow cytometry (BD FACSCalibur; BD Biosciences, NJ, USA) was then used to analyze the cells.

### Sphere formation assay

After the ECSCs had been isolated and digested into single cells, the culture was continued in a 6-well low attachment surface well plate at a density of 5000 cells per well for 7 days under the aforementioned ECSC culture conditions. The cell spheroid was then photographed with a microscope, and its diameter was measured.

### Resistance of carboplatin (CBP)

The half inhibitory concentration (IC50) of endometrial cancer non-stem cells and ECSCs has been previously calculated and found to be 35.81 μg/mL (95%CI = 32.66–39.23 µg/mL) and 63.68 μg/mL (95%CI = 58.34–69.44 µg/mL), respectively. The concentration curve is shown in Figure S1A. Cells were seeded in a 96-well plate with 2 × 10^4^ cells per well. CBP with the IC50 of the endometrial cancer non-stem cells and ECSCs was added to each cell group. After 48 h, CCK8 was added. The absorbance was measured at 450 nm to calculate cell viability.

### Chromatin Immunoprecipitation (ChIP)

The ChIP assay was performed with the ChIP kit (Active Motif, Shanghai, China) according to the manufacturer’s protocol. The main steps were as follows: cell cross-linking and ultrasonic fragmentation of chromatin were performed. The anti-Sox2 antibody (CST), anti-RNA polymerase II (positive control), or normal mouse IgG (negative control) were added to the chromatin solution, respectively. The solution was incubated overnight at 4 °C. The protein and DNA were then de-crosslinked. The DNA was purified and enriched. Primers were designed and synthesized according to the predicted binding sites in the promoter region. qRT-PCR was performed with the primers.

### Tumor xenografts in nude mice

All animal experiments were reviewed and approved by the Scientific Research and New Technology Ethical Committee of the Shengjing Hospital of China Medical University (No. 2018PS136K). Five-week-old female BALB/c athymic nude mice were purchased from HFK Bioscience Co., Ltd. (Beijing, China) and housed in specific pathogen-free conditions. The mice were randomly grouped and subcutaneously injected 1 × 10^6^ endometrial cancer non-stem cells or ECSCs into the axilla of each nude mouse. The growth of the transplanted tumor was recorded every 4 days after injection. The tumor volume was observed continuously for 4 weeks and calculated according to the following formula: tumor volume (mm^3^) = length × width^2^/2. The nude mice were then sacrificed, and their transplanted tumors were removed for other experiments. If the nude mice showed signs of pain during the process of tumor growth, such as significant weight loss, lethargy, or tumor rupture, the nude mice were sacrificed by cervical dislocation.

### Statistical analysis

Data were presented as mean ± standard deviation (SD) of three independent experiments. All statistical analyses were performed using GraphPad Prism 8.0 (LaJolla, CA, USA) and SPSS 19.0 software (Abbott Laboratories, Chicago, IL, USA). Student’s t-tests were used to compare two independent data sets, and the Pearson’s and Spearman’s correlation coefficient analyses were used to determine correlation. Cut-off values were calculated by the receiver operating characteristic (ROC) curve. Survival curves were drawn using the Kaplan–Meier method and compared using log-rank tests. *P* < 0.05 was considered statistically significant.

## Results

### PVT1 is highly expressed in endometrial cancer and promotes malignant behavior of ECCs and ECSCs

**Fig. 1:**
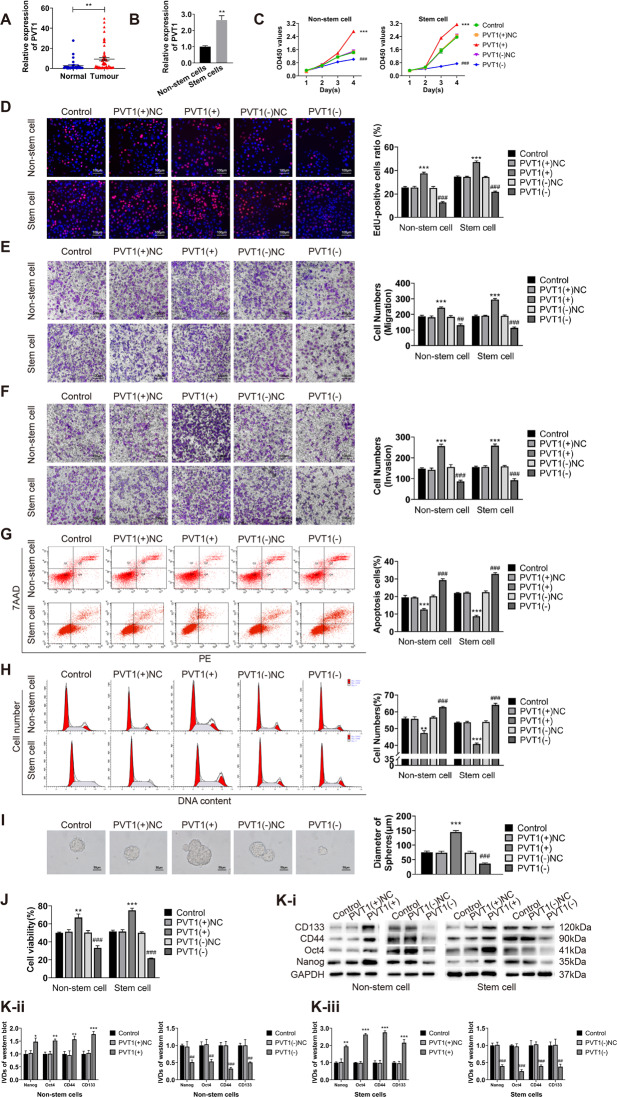
PVT1 is overexpressed in endometrial cancer and promotes the malignant behavior of ECCs and ECSCs. **A** PVT1 expression was evaluated in endometrial cancer tissues (*n* = 55) and normal endometrial tissues (*n* = 30) by qRT-PCR. **B** PVT1 expression was evaluated in endometrial cancer non-stem cells and stem cells by qRT-PCR. **C**, **D** Effects of PVT1 on proliferation in endometrial cancer non-stem cells and stem cells evaluated by CCK8 and EdU assays. **E** Effects of PVT1 on cell migration in endometrial cancer non-stem cells and stem cells evaluated by transwell assays. **F** Effects of PVT1 on cell invasion in endometrial cancer non-stem cells and stem cells evaluated by transwell assays. **G** Effects of PVT1 on the cell cycle (G0/G1 phase) in endometrial cancer non-stem cells and stem cells evaluated by flow cytometry. **H** Effects of PVT1 on cell apoptosis in endometrial cancer non-stem cells and stem cells evaluated by flow cytometry. **I** Effects of PVT1 on cell self-renewal capacity in ECSCs evaluated by sphere formation assays. **J** Effects of PVT1 on resistance of CBP in endometrial cancer non-stem cells and stem cells evaluated by CCK8. **K** Effects of PVT1 on stemness markers (CD133, CD44, Oct4, and Nanog) in endometrial cancer non-stem cells and stem cells evaluated by western blot. Data are presented as the means ± SD (*n* = 3, each group), *P < 0.05, **P < 0.01, ***P < 0.0001 vs. PVT1(+)NC group; ^#^*P* < 0.05, ^##^*P* < 0.01 and ^###^*P* < 0.001 vs. PVT1(-)NC group.

The expression of PVT1 in endometrial cancer tissues and ECSCs were detected by qRT-PCR. Compared with normal endometrial tissue, PVT1 was significantly increased in endometrial cancer tissues (Fig. 1A). Its expression was correlated with various FIGO stages, lymph node metastasis, and LVSI (Table S3). The survival analysis found that patients with high PVT1 expression had worse prognosis (Figure S1B). We also detected the expression of PVT1 in endometrial cancer non-stem cells and stem cells and found that the expression of PVT1 in ECSCs was significantly higher than that in non-stem cells (Fig. 1B). To investigate the biological significance of PVT1 in endometrial cancer, we overexpressed or knockdown PVT1 in endometrial cancer non-stem cells and stem cells (transfection efficiency is shown in Figure S1C). We then examined cell proliferation, migration, invasion, apoptosis, the cell cycle, sphere formation ability, drug resistance, and the expression of stemness markers (CD133, CD44, Oct4, and Nanog). Our results showed that the knockdown of PVT1 inhibited cell proliferation (Fig. 1C, D), migration (Fig. 1E), and invasion (Fig. 1F). Moreover, PVT1 knockdown promoted apoptosis (Fig. 1G) and cell cycle arrest at the G0/G1 phase (Fig. 1H), inhibited the sphere formation ability of cells (Fig. 1I), reduced the resistance of cells to CBP (Fig. 1J), and reduced the expression of the stemness markers (Fig. 1K). In contrast, overexpressing PVT1 in cells resulted in the opposite malignant behavior. These results suggest that PVT1 has a role in promoting malignant behavior and stem cell-like properties in ECCs and ECSCs.

### miR-136 is underexpressed in endometrial cancer and negatively regulated by PVT1

**Fig. 2:**
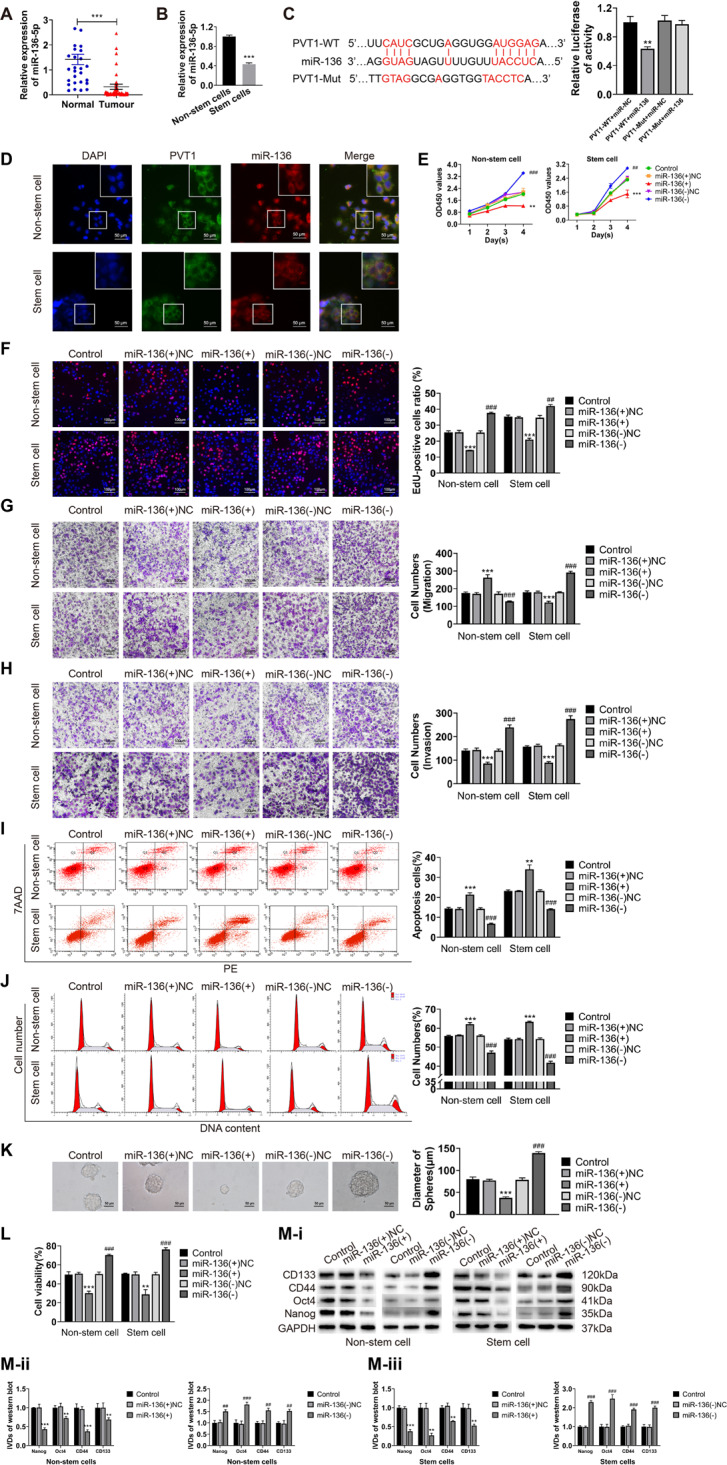
miR-136 is underexpressed in endometrial cancer and inhibit the malignant behavior of ECCs and ECSCs. **A** miR-136 expression was evaluated in endometrial cancer tissues (*n* = 55) and normal endometrial tissues (*n* = 30) by qRT-PCR. **B** miR-136 expression was evaluated in endometrial cancer non-stem cells and stem cells by qRT-PCR. **C** The targeted binding site of PVT1 and miR-36 was validated by dual-luciferase reporter assays. Data are presented as the means ± SD (*n* = 3, each group), *P < 0.05, **P < 0.01, ***P < 0.0001 vs. control group. **D** The co-location of PVT1 and miR-136 in endometrial cancer non-stem cells and stem cells by FISH assays. **E**, **F** Effects of miR-136 on proliferation in endometrial cancer non-stem cells and stem cells evaluated by CCK8 and EdU assays. **G** Effects of miR-136 on cell migration in endometrial cancer non-stem cells and stem cells evaluated by transwell assays. **H** Effects of miR-136 on cell invasion in endometrial cancer non-stem cells and stem cells evaluated by transwell assays. **I** Effects of miR-136 on cell apoptosis in endometrial cancer non-stem cells and stem cells evaluated by flow cytometry. **J** Effects of miR-136 on the cell cycle (G0/G1 phase) in endometrial cancer non-stem cells and stem cells evaluated by flow cytometry. **K** Effects of miR-136 on cell self-renewal capacity in ECSCs evaluated by sphere formation assays. **L** Effects of miR-136 on resistance of CBP in endometrial cancer non-stem cells and stem cells evaluated by CCK8. **M** Effects of miR-136 on stemness markers (CD133, CD44, Oct4, and Nanog) in endometrial cancer non-stem cells and stem cells evaluated by western blot. Data are presented as the means ± SD (*n* = 3, each group), *P < 0.05, **P < 0.01, ***P < 0.0001 vs. miR-136(+)NC group; ^#^*P* < 0.05, ^##^*P* < 0.01, and ^###^*P* < 0.001 vs. miR-136(-)NC group.

The expression of miR-136 in endometrial cancer tissues and ECSCs were detected by qRT-PCR. Compared with normal endometrial tissues, miR-136 was significantly reduced in endometrial cancer tissues (Fig. 2A). Its expression correlated with various FIGO stages and grades (Table S4). The survival analysis found that patients with low miR-136 expression had worse prognosis (Figure S2A). We also detected the expression of miR-136 in endometrial cancer non-stem cells and stem cells and found that the expression of miR-136 in ECSCs was significantly lower than that in non-stem cells (Fig. 2B). We further found that the expression of miR-136 in tissues was negatively correlated with the expression of PVT1 (Pearson’s rank correlation method, *R* = −0.3158, *P* = 0.0188, Figure S2B). The results of the dual-luciferase reporter assay showed that the relative fluorescence activity decreased significantly after the co-transfection of PVT1-WT and mimic-miR-136 (Fig. 2C). FISH assay showed that PVT1 and miR-136 were mainly co-located in the cytoplasm (Fig. 2D).

After overexpressing PVT1 in endometrial cancer non-stem cells and ECSCs, the expression of miR-136 was significantly decreased as found in the qRT-PCR analysis. Conversely, the expression of miR-136 increased after the knockdown of PVT1 (Figure S2C). After the overexpression or knockdown of miR-136 in the two cell groups (transfection efficiency is shown in Figure S2E), the expression of PVT1 also showed an opposite trend (Figure S2D). These results suggest that PVT1 negatively regulates miR-136.

### miR-136 can inhibit the malignant behavior of ECCs and ECSCs

To investigate the biological significance of miR-136 in endometrial cancer, we overexpressed or knocked down miR-136 in endometrial cancer non-stem cells and stem cells. We then examined changes in cell proliferation, migration, invasion, apoptosis, the cell cycle, sphere formation ability, drug resistance, and changes in the expression of stemness markers (CD133, CD44, Oct4, and Nanog). Our results showed that the overexpression of miR-136 could inhibit cell proliferation (Fig. 2E, F), migration (Fig. 2G), and invasion (Fig. 2H). miR-136 overexpression could further promote cell apoptosis (Fig. 2I) and cell cycle arrest at the G0/G1 phase (Fig. 2J), inhibit the sphere formation ability of cells (Fig. 2K), reduce the resistance of cells to CBP (Fig. 2L), and reduce the expression of stemness markers (Fig. 2M). The overexpression of miR-136 therefore resulted in the opposite malignant behavior. These results suggest that miR-136 has a role in suppressing malignant behavior and stem cell-like properties in ECCs and ECSCs.

### miR-136 can restore the inhibitory effect of PVT1 knockdown on the malignant behavior of ECCs and ECSCs

**Fig. 3:**
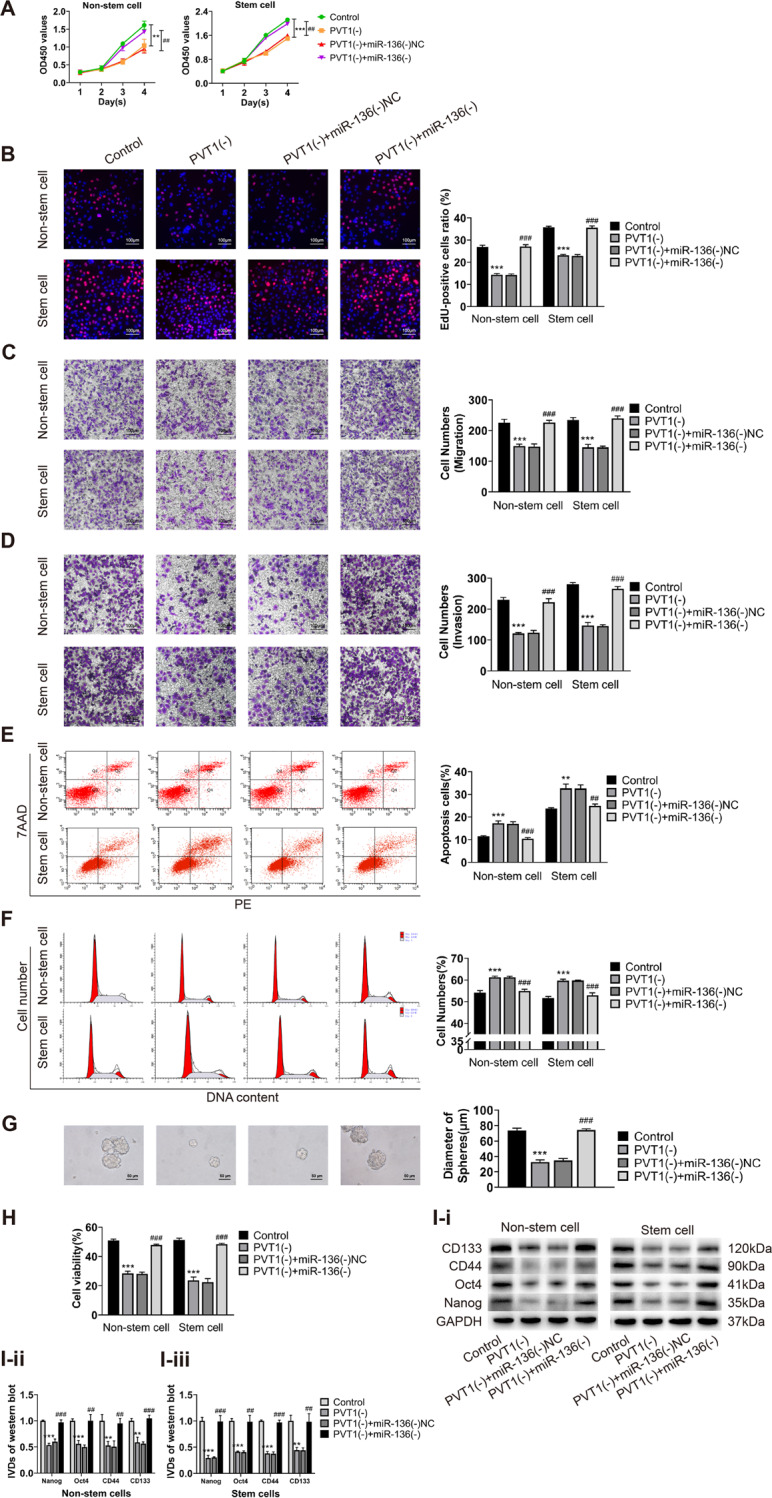
miR-136 can restore the inhibitory effect of PVT1 knockdown on the malignant behavior of ECCs and ECSCs. **A**, **B** Effects of co-transfected PVT1(-) and miR-136(-) on proliferation in endometrial cancer non-stem cells and stem cells evaluated by CCK8 and EdU assays. **C** Effects of co-transfected PVT1(-) and miR-136(-) on cell migration in endometrial cancer non-stem cells and stem cells evaluated by transwell assays. **D** Effects of co-transfected PVT1(-) and miR-136(-) on cell invasion in endometrial cancer non-stem cells and stem cells evaluated by transwell assays. **E** Effects of co-transfected PVT1(-) and miR-136(-) on cell apoptosis in endometrial cancer non-stem cells and stem cells evaluated by flow cytometry. **F** Effects of co-transfected PVT1(-) and miR-136(-) on the cell cycle (G0/G1 phase) in endometrial cancer non-stem cells and stem cells evaluated by flow cytometry. **G** Effects of co-transfected PVT1(-) and miR-136(-) on cell self-renewal capacity in ECSCs evaluated by sphere formation assays. **H** Effects of co-transfected PVT1(-) and miR-136(-) on resistance of CBP in endometrial cancer non-stem cells and stem cells evaluated by CCK8. **I** Effects of co-transfected PVT1(-) and miR-136(-) on stemness markers (CD133, CD44, Oct4, and Nanog) in endometrial cancer non-stem cells and stem cells evaluated by western blot. Data are presented as the means ± SD (*n* = 3, each group), *P < 0.05, **P < 0.01, ***P < 0.0001 vs. control group; ^#^*P* < 0.05, ^##^*P* < 0.01 and ^###^*P* < 0.001 vs. PVT1(-) + miR-136(-)NC group.

To further investigate the interaction between PVT1 and miR-136, we designed rescue experiments. Both PVT1 and miR-136 were knocked down in endometrial cancer non-stem cells and stem cells. Changes in cell proliferation, migration, invasion, apoptosis, the cell cycle, sphere formation ability, drug resistance, and changes in the expression of stemness markers (CD133, CD44, Oct4, and Nanog) were analyzed. Our results showed that, compared to cells with PVT1 knockout alone, the simultaneous knockdown of PVT1 and miR-136 restored suppressed cell proliferation, migration, and invasion (Fig. 3A–D); restored promoted apoptosis and cell cycle arrest at the G0/G1 phase (Fig. 3E, F); and restored the reduced cell sphere formation ability, CBP resistance, and the expression of stemness markers (Fig. 3G–I). The results of the rescue experiments indicated that PVT1 and miR-136 had functional targeted binding in ECCs and ECSCs, and reducing miR-136 expression could reverse the antitumor effect of PVT1 knockdown.

### Sox2 is highly expressed in endometrial cancer and participates in PVT1/miR-136 regulation of malignant behavior of ECCs and ECSCs

**Fig. 4:**
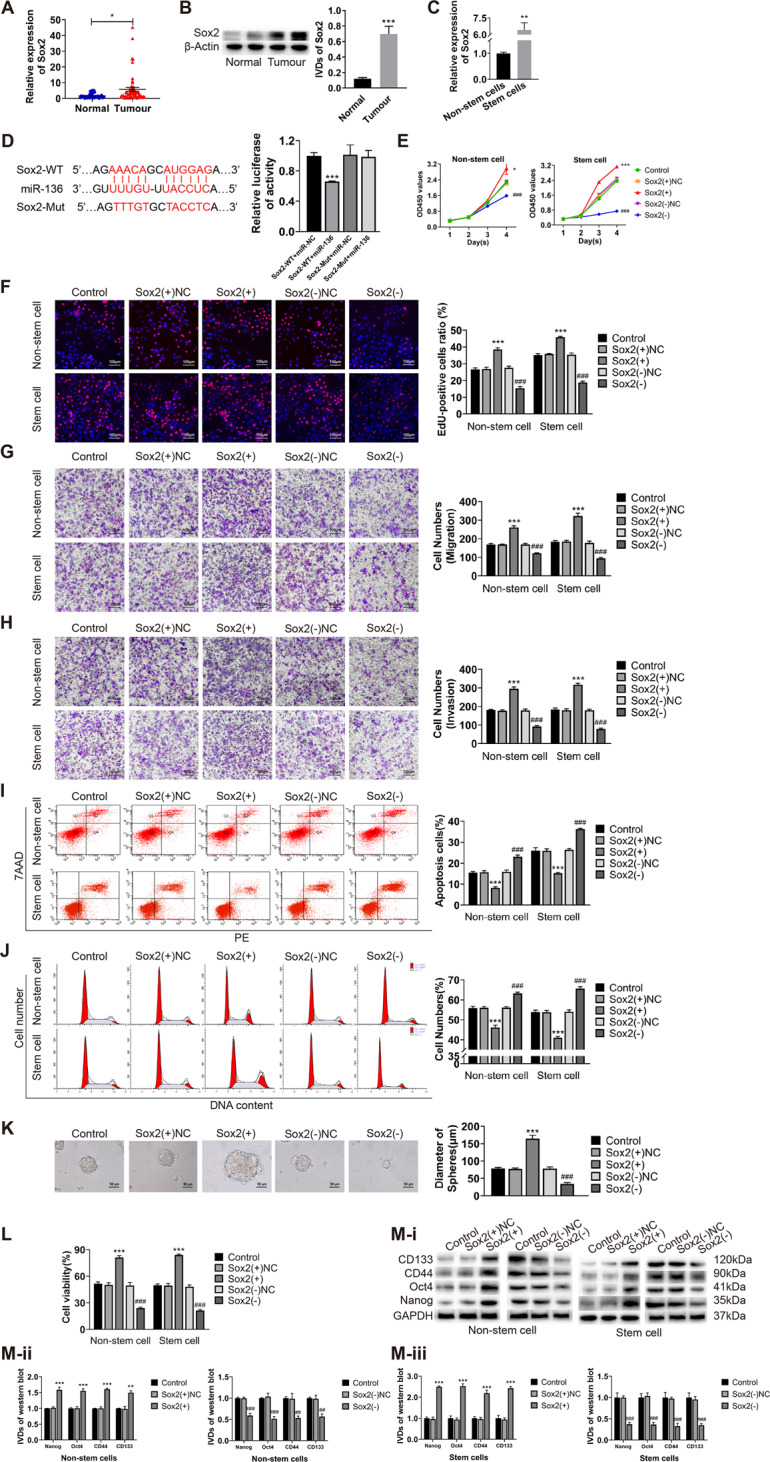
Sox2 is highly expressed in endometrial cancer and promote malignant behavior of ECCs and ECSCs by participating in PVT1/miR-136 regulation. **A**, **B** Sox2 expression was evaluated in endometrial cancer tissues (*n* = 55) and normal endometrial tissues (*n* = 30) by qRT-PCR and western blot. **C** Sox2 expression was evaluated in endometrial cancer non-stem cells and stem cells by qRT-PCR. **D** The targeted binding site of Sox2 and miR-36 was validated by dual-luciferase reporter assays. Data are presented as the means ± SD (*n* = 3, each group), *P < 0.05, **P < 0.01, ***P < 0.0001 vs. control group. **E**, **F** Effects of Sox2 on proliferation in endometrial cancer non-stem cells and stem cells evaluated by CCK8 and EdU assays. **G** Effects of Sox2 on cell migration in endometrial cancer non-stem cells and stem cells evaluated by transwell assays. **H** Effects of Sox2 on cell invasion in endometrial cancer non-stem cells and stem cells evaluated by transwell assays. **I** Effects of Sox2 on cell apoptosis in endometrial cancer non-stem cells and stem cells evaluated by flow cytometry. **J** Effects of Sox2 on the cell cycle (G0/G1 phase) in endometrial cancer non-stem cells and stem cells evaluated by flow cytometry. **K** Effects of Sox2 on cell self-renewal capacity in ECSCs evaluated by sphere formation assays. **L** Effects of Sox2 on resistance of CBP in endometrial cancer non-stem cells and stem cells evaluated by CCK8. **M** Effects of Sox2 on stemness markers (CD133, CD44, Oct4, and Nanog) in endometrial cancer non-stem cells and stem cells evaluated by western blot. Data are presented as the means ± SD (*n* = 3, each group), *P < 0.05, **P < 0.01, ***P < 0.0001 vs. Sox2(+)NC group; ^#^*P* < 0.05, ^##^*P* < 0.01 and ^###^*P* < 0.001 vs. Sox2(-)NC group.

According to the qRT-PCR and western blot results, we found that the expression of Sox2 in endometrial cancer tissues was higher than that in normal endometrial tissues (Fig. 4A, B). The expression of Sox2 in ECSCs was also significantly higher than that in non-stem cells (Fig. 4C). Moreover, the expression of Sox2 in tissues was negatively correlated with the expression of miR-136 (Pearson’s rank correlation method, *R* = −0.2675, *P* = 0.0483, Figure S3A). The results of the dual-luciferase reporter assay showed that the relative fluorescence activity decreased significantly after the co-transfection of Sox2-WT and mimic-miR-136 (Fig. 4D), and the binding site was the same as that of PVT1 and miR-136. After overexpressing PVT1 in endometrial cancer non-stem cells and ECSCs, the expression of Sox2 was significantly decreased as noted in the qRT-PCR analysis, while the expression of Sox2 increased after PVT1 knockdown (Figure S3B). The expression of Sox2 decreased following miR-136 overexpression in both cell groups, whereas it increased after miR-136 knockdown (Figure S3C). In addition, compared to cells with PVT1 knockout alone, the simultaneous knockdown of PVT1 and miR-136 restored the expression of Sox2 (Figure S3D). These results indicate that PVT1 positively regulates Sox2; miR-136 negatively regulates Sox2; and the interaction between PVT1 and miR-136 can regulate the expression of Sox2.

### Sox2 can promote malignant behavior of ECCs and ECSCs

Considering that Sox2 may play a role in PVT1-miR-136 regulating the malignant behavior of ECCs and ECSCs, we overexpressed and knocked down Sox2 in both cell groups (transfection efficiency is shown in Figure S3E). Changes in cell proliferation, migration, invasion, apoptosis, the cell cycle, sphere formation ability, drug resistance, and the expression of stemness markers (CD133, CD44, Oct4, and Nanog) were then examined. Our results showed that Sox2 knockdown could inhibit cell proliferation (Fig. 4E, F), migration (Fig. 4G), and invasion (Fig. 4H); promote cell apoptosis (Fig. 4I) and cell cycle arrest in the G0/G1 phase (Fig. 4J); inhibit the sphere formation ability of cells (Fig. 4K); and reduce the resistance of cells to CBP (Fig. 4L) and the expression of the stemness markers (Fig. 4M). The overexpression of Sox2 resulted in the opposite malignant behavior. These results suggest that Sox2 plays a role in promoting malignant behavior and stem cell-like properties in ECCs and ECSCs.

### Sox2 can restore the inhibitory effect of miR-136 on malignant behavior of ECCs and ECSCs

**Fig. 5:**
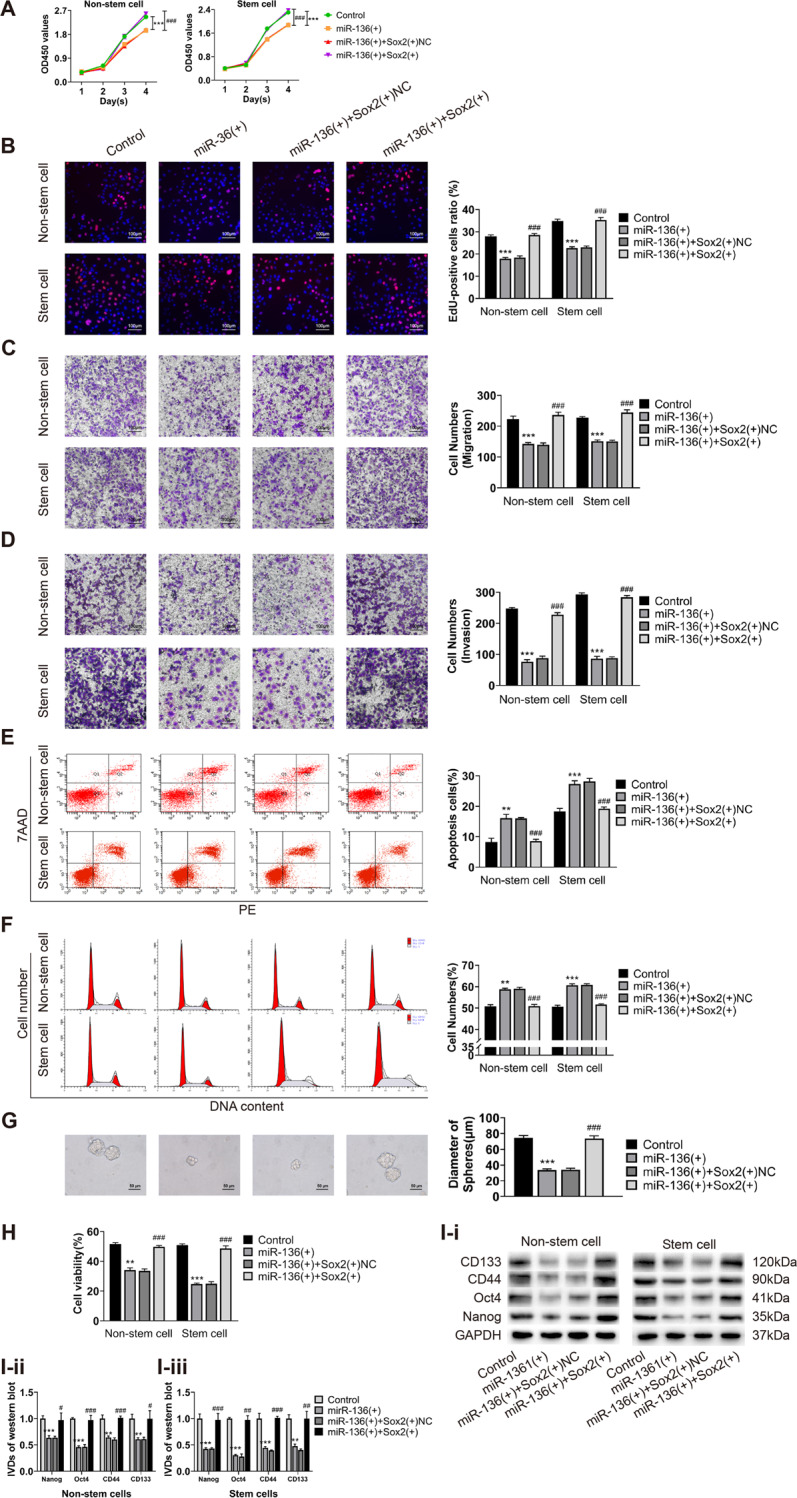
Sox2 can restore the inhibitory effect of miR-136 on malignant behavior of ECCs and ECSCs. **A**, **B** Effects of co-transfected miR-136 and Sox2 on proliferation in endometrial cancer non-stem cells and stem cells evaluated by CCK8 and EdU assays. **C** Effects of co-transfected PVT1 and miR-136 on cell migration in endometrial cancer non-stem cells and stem cells evaluated by transwell assays. **D** Effects of co-transfected miR-136 and Sox2 on cell invasion in endometrial cancer non-stem cells and stem cells evaluated by transwell assays. **E** Effects of co-transfected miR-136 and Sox2 on cell apoptosis in endometrial cancer non-stem cells and stem cells evaluated by flow cytometry. **F** Effects of co-transfected miR-136 and Sox2 on the cell cycle (G0/G1 phase) in endometrial cancer non-stem cells and stem cells evaluated by flow cytometry. **G** Effects of co-transfected miR-136 and Sox2 on cell self-renewal capacity in ECSCs evaluated by sphere formation assays. **H** Effects of co-transfected miR-136 and Sox2 on resistance of CBP in endometrial cancer non-stem cells and stem cells evaluated by CCK8. **I** Effects of co-transfected miR-136 and Sox2 on stemness markers (CD133, CD44, Oct4, and Nanog) in endometrial cancer non-stem cells and stem cells evaluated by western blot. Data are presented as the means ± SD (*n* = 3, each group), *P < 0.05, **P < 0.01, ***P < 0.0001 vs. control group; ^#^*P* < 0.05, ^##^*P* < 0.01 and ^###^*P* < 0.001 vs. miR-136(+)+Sox2(+)NC group.

To further investigate the interaction between miR-136 and Sox2, we designed rescue experiments. Both miR-136 and Sox2 were overexpressed in endometrial cancer non-stem cells and stem cells. Changes in cell proliferation, migration, invasion, apoptosis, the cell cycle, sphere formation ability, drug resistance, and changes in the expression of stemness markers (CD133, CD44, Oct4, and Nanog) were examined. Our results showed that the simultaneous overexpression of miR-136 and Sox2 restored suppressed cell proliferation, migration, and invasion compared to cells with upregulated miR-136 alone (Fig. 5A–D); restored promoted apoptosis and cell cycle arrest in the G0/G1 phase (Fig. 5E, F); and restored the reduced cell sphere formation ability, CBP resistance, and the expression of the stemness markers (Fig. 5G–I). The results of the rescue experiments showed that miR-136 had a functional target binding to Sox2 in ECCs and ECSCs, and elevated Sox2 expression could reverse the antitumor effect of miR-136.

### UPF1 is the direct target of Sox2 to promote malignant behavior in ECCs and ECSCs

**Fig. 6:**
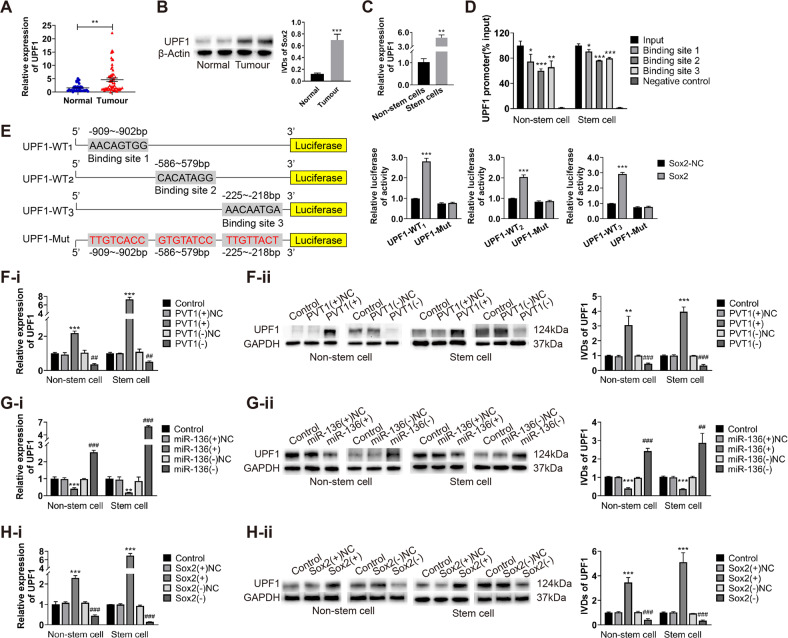
UPF1 is the direct target of Sox2 to promote malignant behavior in ECCs and ECSCs. **A**, **B** UPF1 expression was evaluated in endometrial cancer tissues (*n* = 55) and normal endometrial tissues (*n* = 30) by qRT-PCR and western blot. **C** UPF1 expression was evaluated in endometrial cancer non-stem cells and stem cells by qRT-PCR. **D** The binding of Sox2 and UPF1 was detected by ChIP. **E** The targeted binding sites of Sox2 and UPF1 were validated by dual-luciferase reporter assays. Data are presented as the means ± SD (*n* = 3, each group), *P < 0.05, **P < 0.01, ***P < 0.0001 vs. control group. **F** Changes in the expression of UPF1 were detected by qRT-PCR and western blot after PVT1 was regulated. Data are presented as the means ± SD (*n* = 3, each group), *P < 0.05, **P < 0.01, ***P < 0.0001 vs. PVT1(+)NC group; ^#^*P* < 0.05, ^##^*P* < 0.01 and ^###^*P* < 0.001 vs. PVT1(-)NC group. **G** Changes in the expression of UPF1 were detected by qRT-PCR and western blot after miR-136 was regulated. Data are presented as the means ± SD (*n* = 3, each group), *P < 0.05, **P < 0.01, ***P < 0.0001 vs. miR-136(+)NC group; ^#^*P* < 0.05, ^##^*P* < 0.01 and ^###^*P* < 0.001 vs. miR-136(-)NC group. **H** Changes in the expression of UPF1 were detected by qRT-PCR and western blot after Sox2 was regulated. Data are presented as the means ± SD (*n* = 3, each group), *P < 0.05, **P < 0.01, ***P < 0.0001 vs. Sox2(+)NC group; ^#^*P* < 0.05, ^##^*P* < 0.01 and ^###^*P* < 0.001 vs. Sox2(-)NC group.

According to previous studies, UPF1 promotes malignant behavior and stem cell properties in ECCs and ECSCs. The expression of UPF1 in endometrial cancer tissues was higher than that in normal endometrium (Fig. 6A, B) and higher in ECSCs than in non-stem cells (Fig. 6C). According to the JASPAR database (http://jaspar.genereg.net/cgi-bin/jaspar_db.pl), we predicted that Sox2 had three binding sites in the promoter region of UPF1, which may act as transcription factors to target and regulate the cancer-promoting effect of UPF1. We therefore examined the relationship between Sox2 and UPF1. Through ChIP experiments, we found that Sox2 could bind to all three sites (Fig. 6D). To further investigate the binding sites and the role of Sox2 on UPF1 expression, we performed a dual-luciferase reporter assay. The results showed that after co-transfection of the wild-type dual-luciferase vector containing the binding site of the UPF1 promoter region and Sox2, the fluorescence value more than doubled compared with the co-transfection of wild-type dual-luciferase vector and Sox2-NC. However, the fluorescence value did not increase after the mutant dual-luciferase vector was co-transfected with Sox2 (Fig. 6E). After regulating the expression of PVT1 in ECCs and ECSCs, the expression of UPF1 showed the same trend as PVT1 (Fig. 6F). After regulating the expression of miR-136, the expression of UPF1 exhibited the opposite trend to that of miR-136 (Fig. 6G). After regulating the expression of Sox2, the expression of UPF1 showed the same trend as Sox2 (Fig. 6H). Moreover, our results showed that the simultaneous knockdown of PVT1 and miR-136 restored the expression of UPF1 compared to cells with PVT1 knockout alone (Figure S4A), and compared to cells with upregulated miR-136 alone, overexpressed both miR-136 and Sox2 restored the expression of UPF1 (Figure 4SB). These results suggest that the PVT1/miR-136/Sox2 axis can regulate the transcription of UPF1 to influence the malignant behavior of ECCs and ECSCs.

### PVT1 downregulation combined with miR-136 upregulation can significantly inhibit the growth of xenograft tumors in nude mice

**Fig. 7:**
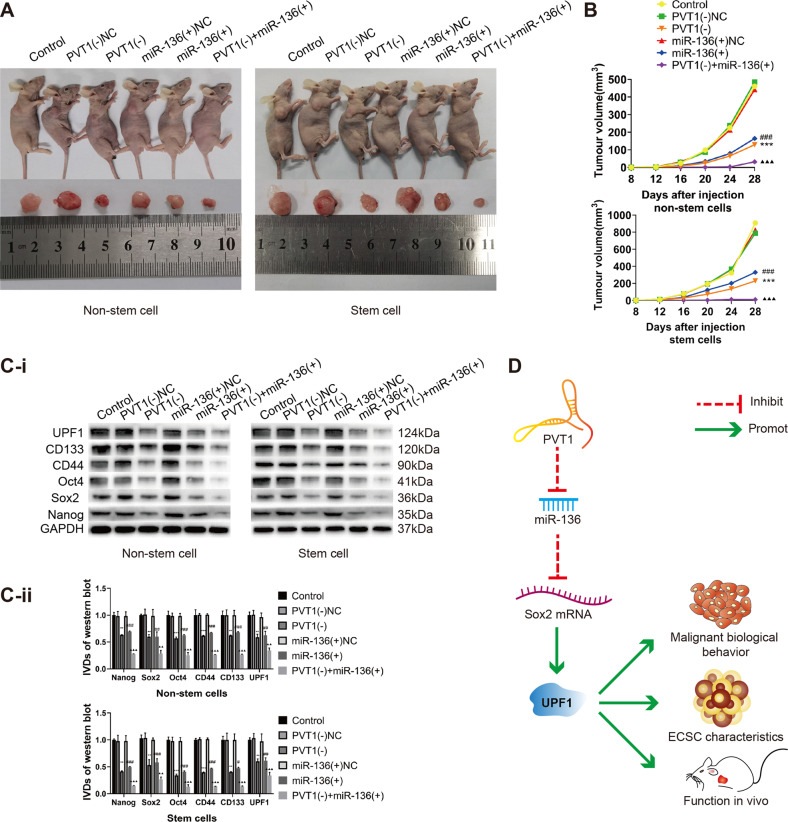
Downregulation of PVT1 combined with upregulation of miR-136 can significantly inhibit the growth of xenograft tumors in nude mice. **A** Tumor-bearing nude mice and one tumor sample from each group are shown. **B** The volume of each group of tumors at different observation time. **C** Western blot was used to detect the expression of Sox2, UPF1, and stem cell markers in nude mouse tumors formed by endometrial cancer non-stem cells and stem cells. Data are presented as the means ± SD (*n* = 3, each group), *P < 0.05, **P < 0.01, ***P < 0.0001 vs. PVT1(-)NC group; ^#^*P* < 0.05, ^##^*P* < 0.01 and ^###^*P* < 0.001 vs. miR-136(-)NC group; ^▲^*P* < 0.05, ^▲▲^*P* < 0.01, ^▲▲▲^*P* < 0.0001 vs. miR-136(-) group. **D** Schematic diagram of the PVT1/miR-136/Sox2/UPF1 axis mechanism.

We determined the effects of PVT1 downregulation, miR-136 upregulation, and the downregulation of PVT1 combined with the upregulation of miR-136 on the growth of xenografts in nude mice. The results showed that PVT1 downregulation or miR-136 upregulation could inhibit the growth of xenograft tumors in nude mice, while PVT1 downregulation combined with miR-136 upregulation significantly inhibited the growth of xenograft tumors in nude mice more than the regulation of PVT1 or miR-136 alone (Fig. 7A, B). Moreover, in the xenografts with downregulated PVT1 and upregulated miR-136, the expressions of Sox2, UPF1, and cell stemness markers were decreased. The expression of miR-136 was significantly lower than when PVT1 or miR-136 were regulated alone (Fig. 7C).

## Discussion

In recent years, endometrial cancer has threatened women’s health worldwide. The intractable diseases, such as advanced and recurrent endometrial cancer, are especially concerning. The TSC theory has opened a new field of research for endometrial cancer. TSCs maintain the vitality of tumor cell populations through self-renewal and infinite proliferation. The movement and migration ability of TSCs makes the metastasis of tumor cells possible. Similar to ordinary stem cells, TSCs have molecular pumps that can expel chemotherapeutic drugs. They are also less sensitive to chemotherapeutic drugs and more resistant to chemotherapy and radiation therapy than differentiated cells. As a result, tumors often recur within a period of time after conventional treatments have eradicated most of the normal tumor cells. Although some researchers have found that the occurrence, development, metastasis, and recurrence of endometrial cancer are closely related to TSCs with stem cell potential [26], the specific mechanism of action is not clear. Therefore, we carried out in-depth investigations of endometrial cancer non-stem cells and ECSCs to uncover these mechanisms.

Our results showed that lncRNA-PVT1 expression was elevated in endometrial cancer tissues and ECSCs. Patients with high PVT1 expression had worse prognosis, a later FIGO stage, and were associated with lymphatic metastasis and LVSI. PVT1 promoted the proliferation, migration, and invasion of endometrial cancer non-stem cells and stem cells, inhibited cell apoptosis, stimulated the cell cycle, promoted the self-renewal of ECSCs, reduced the sensitivity to CBP, increased the expression of stem cell markers, and promoted tumor growth. In the 1980s, PVT1 was discovered and named as a MYC activator [27], and it has presented a tumor-promoting effect in a variety of malignant tumors. For example, PVT1 is upregulated in cervical cancer and promotes the proliferation, migration, and invasion of cervical cancer cells [28]. Reducing the expression of PVT1 can increase the apoptosis and cisplatin sensitivity of cervical cancer cells [29]. PVT1 is highly expressed in epithelial ovarian cancer tissues [30], and the high expression of PVT1 is associated with poor prognosis. Multivariate analyses have proven that PVT1 can be used as an independent prognostic factor for the recurrence and survival of epithelial ovarian cancer [31]. ZOU et al. [32] reported that PVT1 affects the proliferation and invasion of ovarian cancer cells by affecting the expression of SOX2, and other studies have found that PVT1 can enhance the resistance of ovarian cancer to cisplatin [33, 34]. Our findings confirmed that the role of PVT1 in endometrial cancer non-stem cells and ECSCs is similar to that in other tumors and thereby provided a solid foundation for studying the function of PVT1 in endometrial cancer.

As a lncRNA, PVT1 can upregulate the expression of target genes by acting as a competitive endogenous RNA (ceRNA) for miRNA, thereby regulating the malignant biological behavior of tumors. In endometrial cancer, PVT1 can bind to miRNAs, such as miR-612, miR-195-5p, and miR-508-5p, and regulates downstream target genes to promote cancer [9, 10, 25]. In this study, the bioinformatics system predicted and experimentally verified that there is a conserved binding site between the MRE sequence of PVT1 and miR-136. The expression of miR-136 in endometrial cancer tissues and ECSCs was found to be low, and it was negatively correlated with the expression of PVT1. The low expression of miR-136 was associated with poorer prognosis, later stage disease, and poorer differentiation in patients. In ovarian cancer, miR-136 enhances paclitaxel sensitivity and inhibits tumor progression by targeting CBX2 [35]. In triple-negative breast cancer, circPTK2 directly regulates the NFBI and AKT/PI3K pathways through miR-136 to promote cancer progression [36]. In recent years, some researchers have found that miR-136 is involved in the regulation of the biological behavior of ECCs, but the specific role and regulatory mechanism are still unclear [37–39]. In this experiment, it was confirmed that miR-136 is negatively regulated by PVT1, which can inhibit the cell proliferation, migration, and invasion of endometrial cancer non-stem cells and ECSCs; enhance cell apoptosis; block the cell cycle; reduce stem cell self-renewal, chemotherapy, and TSC characteristics, such as drug resistance and stem cell marker expression; and restore the effect of PVT1 on endometrial cancer non-stem cells and ECSCs. These results suggest that PVT1 affects the malignant behavior of endometrial cancer by targeting miR-136.

We predicted that the binding site sequence of Sox2 mRNA-3’ UTR and miR-136 were identical to those of PVT1 and miR-136; therefore, we hypothesized that PVT1 binds miR-136 through competition with Sox2 mRNA to regulate the expression of Sox2. Sox2 is a member of the SRY-related HMGbox (Sox) gene family. The common feature of this family is a highly conserved high mobility group box (HMG-box) DNA binding domain, which plays an important role in regulating embryonic and tissue development, stem cell pluripotency, proliferation, maintaining undifferentiated state, cell division, and determining cell fate. Sox2 can not only directly bind to DNA targets to regulate the expression of related genes, but also form complexes with other proteins as a transcriptional activator to maintain the undifferentiated state and self-renewal of embryonic stem cells [40]. This is considered essential in maintaining the biological characteristics of stem cells. As a stem cell transcription factor, Sox2 can affect the proliferation, metastasis, invasion, apoptosis, drug resistance, and TSC properties of various tumor cells [41–43]. It has further emerged as an attractive target in cancer therapy due to its role in cancer progression and therapy resistance [44]. Our results showed that the expression of Sox2 was significantly increased in endometrial cancer tissues and ECSCs and was negatively correlated with the expression of miR-136. The results of the dual-luciferase reporter assay showed that Sox2 and miR-136 could bind at the predicted site. Sox2 could also be positively regulated by PVT1 or negatively regulated by miR-136. In further experiments, we found that Sox2 can promote the proliferation, migration, and invasion of endometrial cancer non-stem cells and ECSCs, reduce apoptosis and cell cycle arrest, enhance cell self-renewal ability and chemotherapeutic drug resistance, and enhance stem cell marker expression. In addition, Sox2 was able to rescue the inhibitory effect of miR-136 on the malignant behavior and stem cell properties of endometrial cancer non-stem cells and ECSCs. This indicates that Sox2 is a target gene of PVT1 to regulate miR-136 in endometrial cancer non-stem cells and ECSCs and has a tumor-promoting effect on endometrial cancer.

As a common transcription factor, Sox2 may play a role in DNA transcription in endometrial cancer non-stem cells and ECSCs. Through ChIP and dual-luciferase reporter assays, we found that Sox2 acts as a transcription factor for UPF1, and the overexpression of Sox2 in endometrial cancer non-stem cells and ECSCs can increase the expression of UPF1. UPF1 is an RNA-binding protein that can undergo NMD; staufen-mediated mRNA decay; replication-dependent histone mRNA decay; glucocorticoid receptor-mediated mRNA decay; regnase 1-mediated mRNA decay; and tudor-staphylococcal/micrococcal-like nuclease-mediated microRNA decay. Moreover, UPF1 can regulate the malignant behavior of tumors [45, 46], maintain stem cell stemness, and inhibit differentiation [23, 24, 47]. The expression of UPF1 in endometrial cancer tissues is higher than that in adjacent tissues, which can promote the growth and progression of endometrial cancer, increase the activity of the mTOR pathway, inhibit autophagy [48], and promote the malignant behavior and stemness of ECSCs [25]. Through our experiments, we identified UPF1 as a downstream gene target of the PVT1/miR-136/Sox2 axis that regulates the malignant behavior and stemness of ECCs.

In conclusion, this study revealed for the first time that PVT1 competes with Sox2 mRNA-3’ UTR to bind miR-136 and increase Sox2 expression. This enhances UPF1 transcription and promotes the malignant biological behavior and TSC characteristics of endometrial cancer. A schematic diagram of the mechanism is shown in Fig. 7D. The PVT1/miR-136/Sox2/UPF1 axis provides a promising novel approach for the treatment of endometrial cancer, especially refractory endometrial cancer.

## Supplementary information

Table S1-S4

Figure S1

Figure S2

Figure S3

Figure S4

Supplementary figure legends

Original Data File

RELATED MANUSCRIPT FILE

## Data Availability

The datasets used and/or analyzed during the current study are available from the corresponding author on reasonable request.
