# Supplementary material for: PVT1/miR-136/Sox2/UPF1 axis regulates the malignant phenotypes of endometrial cancer stem cells
Source: Cell Death Dis. 2023 Mar 3;14(3):177. doi: 10.1038/s41419-023-05651-0 (PMC9984375; doi:10.1038/s41419-023-05651-0)
Supplement: Supplementary file 1 — Table S1-S4 [file 41419_2023_5651_MOESM1_ESM.docx]

Table S1. Primer sequences for qRT-PCR

| Name | Sequence |
| --- | --- |
| PVT1 | F: GCTGTGGCTGAATGCCTCAT |
|  | R: TTCACCAGGAAGAGTCGGGG |
| miR-136 | F: GCGCATCATCGTCTCAAATGAGTCT |
| Sox2 | F: ACGCTCATGAAGAAGGATAAGT |
|  | R: GAGCTGGTCATGGAGTTGTAC |
| UPF1 | F: GAAGCTGGTCAACACTATCAAC |
|  | R: GTCATGGGTCTGGAAGTACATG |
| GAPDH | F: GCACCGTCAAGGCTGAGAAC |
|  | R: TGGTGAAGACGCCAGTGGA |
| U6 | F: CGGGTTTGTTTTGCATTTCT |
|  | R: AGTCCCAGCATGAACAGCTT |
| UPF1  -binding site 1 | F: CTCCTAGCATGCACTAAACTGTAGGAA |
|  | R: CCCCTTAAACTGTGCCTTCTTGG |
| UPF1  -binding site 2 | F: CGCACCGGGCTCCATATATATCAG |
|  | R: AGCCTGGGTGACAGAGATCCTATG |
| UPF1  -binding site 3 | F: CCACCACAGACCATGTGCTT |
|  | R: TTGCTGCTGTTCCTGATGCC |

Table S2. Sequences of various vectors

| Name | Sequence |
| --- | --- |
| LV-PVT1-RNAi | 5’ -GCAGCTTATTATAGACTTA-3’ |
| LV-NC | 5’ -TTCTCCGAACGTGTCACGT-3’ |
| miR-136 mimic | Sense: 5’ -ACUCCAUUUGUUUUGAUGAUGGA-3’  Antisense: 5’ -CAUCAUCAAAACAAAUGGAGUUU-3’ |
| mimic-nc | Sense: 5’ -UUCUCCGAACGUGUCACGUTT-3’  Antisense: 5’ -ACGUGACACGUUCGGAGAATT-3’ |
| miR-136 inhibitor | 5’ -UCCAUCAUCAAAACAAAUGGAGU-3’ |
| Inhibitor-nc | 5’ -CAGUACUUUUGUGUAGUACAA-3’ |
| sh-Sox2 | ----5’-GGACAGTTACGCGCACATGAATTCA-A  3’ -TTCCTGTCAATGCGCGTGTACTTAGAG |
| sh-NC | --5’ -GTTCTCCGAACGTGTCACGTCAAG-A  3’ -TTAAGAGGCTTGCACAGTGCATTAG |

Table S3. Relationship of PVT1 expression with the clinical characteristics of endometrial carcinoma

| Clinical characteristics | N | Relative expression of PVT1 | P-values |
| --- | --- | --- | --- |
| Age | | | 0.999 |
| ＜60 | 27 | 9.2821±10.9583 |  |
| ≥60 | 28 | 9.2865±13.8251 |  |
| FIGO stage | | | **0.004** |
| I-II | 42 | 6.6484±10.8552 |  |
| III-IV | 13 | 17.8005±13.5921 |  |
| Grade | | | 0.087 |
| G1 | 43 | 7.7756±10.9099 |  |
| G2-3 | 12 | 14.6907±16.0542 |  |
| Invasion depth | | | 0.458 |
| Superficial | 33 | 8.2611±10.6151 |  |
| Deep | 22 | 10.8192±14.7853 |  |
| LVSI | | | **0.025** |
| No | 38 | 7.118±10.641 |  |
| Yes | 17 | 15.6287±15.1852 |  |
| Lymphatic metastasis | | | **0.042** |
| No | 42 | 7.4086±11.0608 |  |
| Yes | 13 | 15.3445±14.837 |  |
| Distal metastasis | | | 0.882 |
| No | 47 | 9.1806±12.0162 |  |
| Yes | 8 | 9.8939±15.2945 |  |

Table S4. Relationship of miR-136 expression with the clinical characteristics of endometrial carcinoma

| Clinical characteristics | N | Relative expression of miR-136 | P-values |
| --- | --- | --- | --- |
| Age | | | 0.757 |
| ＜60 | 27 | 0.2792±0.5981 |  |
| ≥60 | 28 | 0.2354±0.4383 |  |
| FIGO stage | | | **0.002** |
| I-II | 42 | 0.3256±0.5774 |  |
| III-IV | 13 | 0.035±0.0268 |  |
| Grade | | | **0.02** |
| G1 | 43 | 0.3068±0.5742 |  |
| G2-3 | 12 | 0.0783±0.1275 |  |
| Invasion depth | | |  |
| Superficial | 33 | 0.2322±0.392 | 0.668 |
| Deep | 22 | 0.2941±0.6742 |  |
| LVSI | | | 0.688 |
| No | 38 | 0.2735±0.5227 |  |
| Yes | 17 | 0.2083±0.5219 |  |
| Lymphatic metastasis | | | 0.822 |
| No | 42 | 0.2658±0.5152 |  |
| Yes | 13 | 0.2283±0.5491 |  |
| Distal metastasis | | | 0.535 |
| No | 47 | 0.2751±0.5562 |  |
| Yes | 8 | 0.1504±0.1598 |  |
