# Supplementary figures and images for: PVT1/miR-136/Sox2/UPF1 axis regulates the malignant phenotypes of endometrial cancer stem cells
Source: Cell Death Dis. 2023 Mar 3;14(3):177. doi: 10.1038/s41419-023-05651-0 (PMC9984375; doi:10.1038/s41419-023-05651-0)

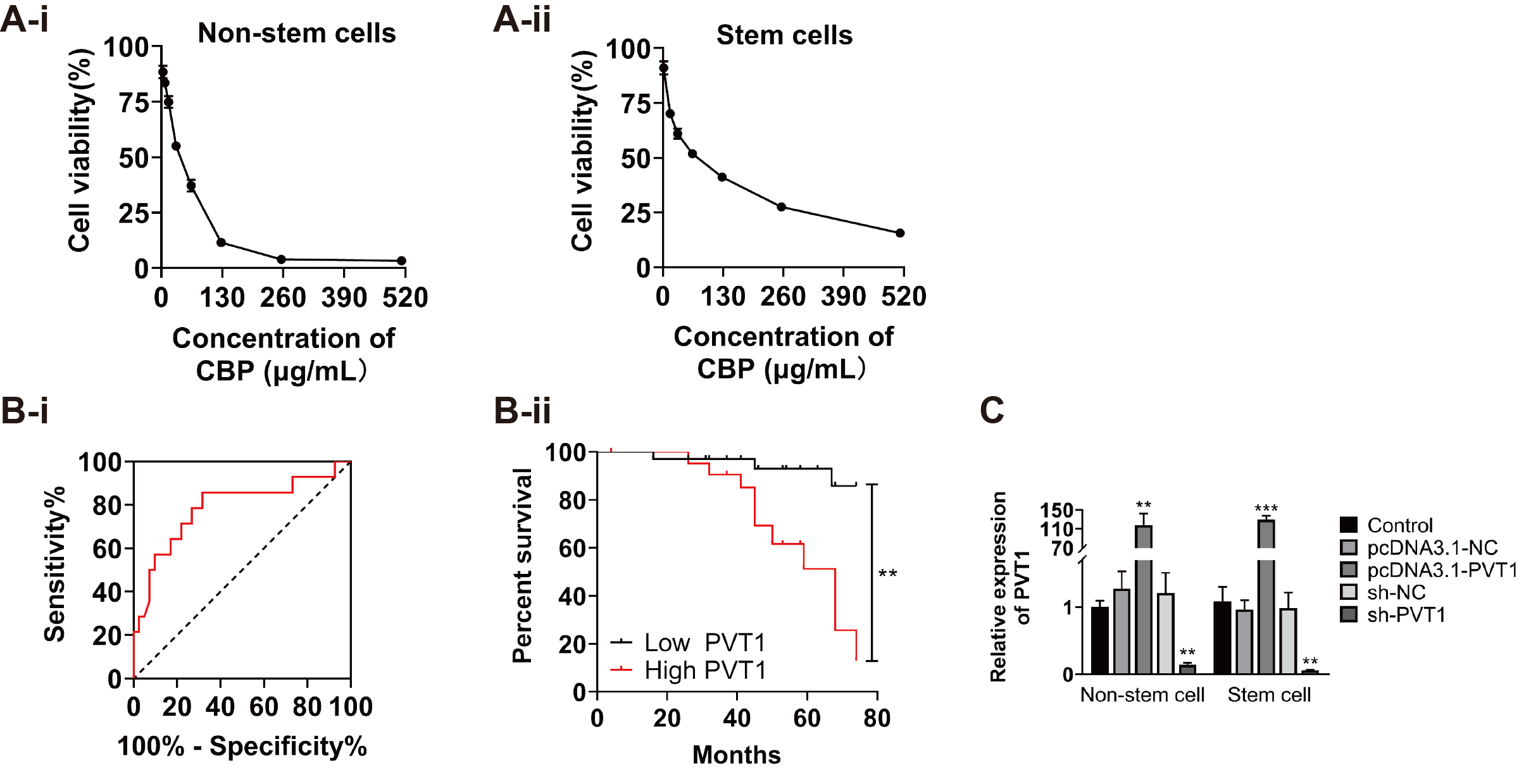

Supplement: Supplementary file 2 — Figure S1 [file 41419_2023_5651_MOESM2_ESM.png]

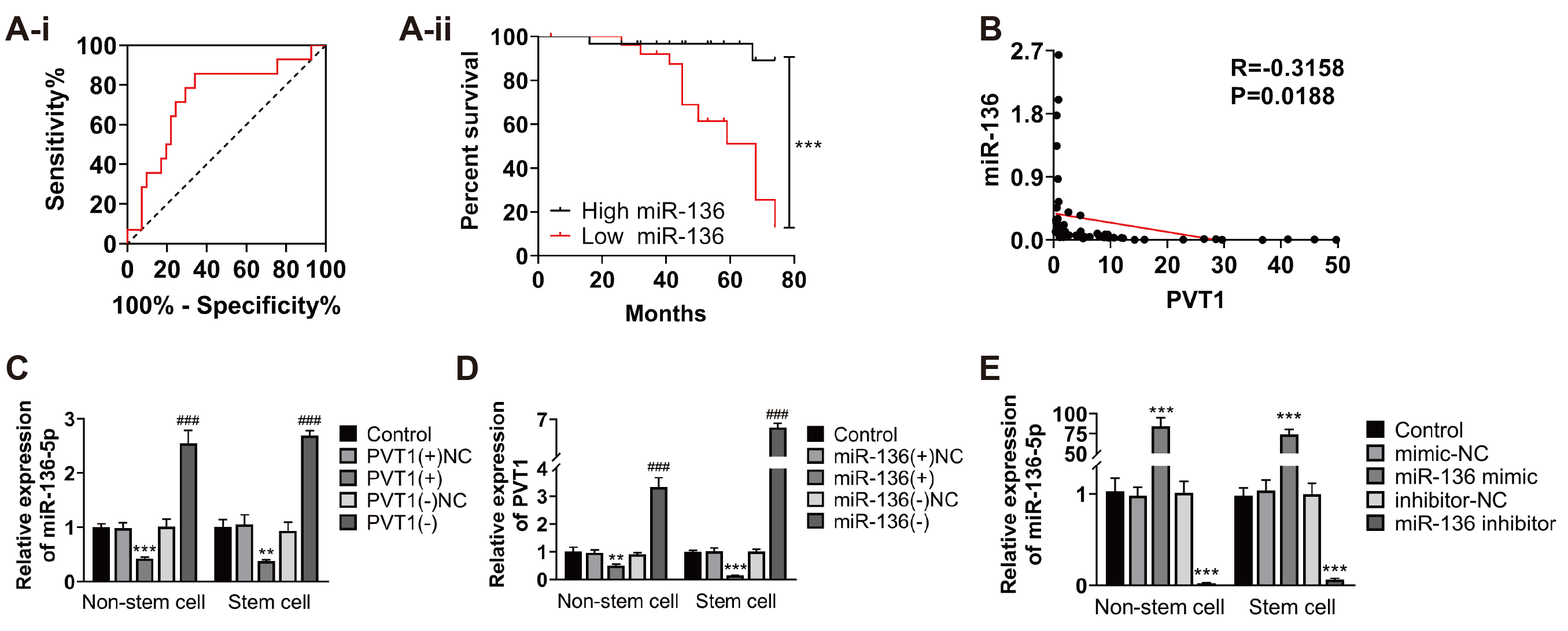

Supplement: Supplementary file 3 — Figure S2 [file 41419_2023_5651_MOESM3_ESM.png]

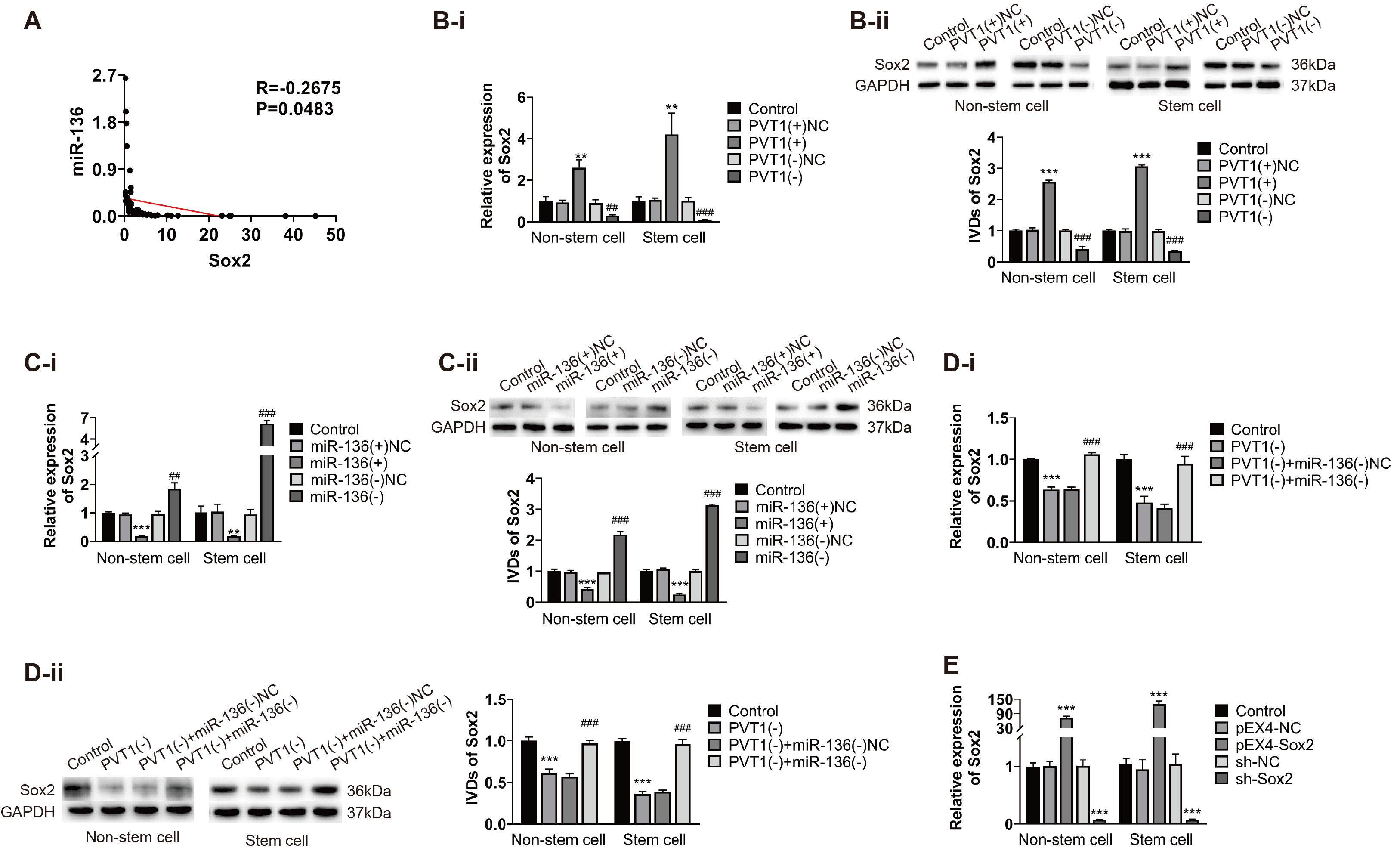

Supplement: Supplementary file 4 — Figure S3 [file 41419_2023_5651_MOESM4_ESM.png]

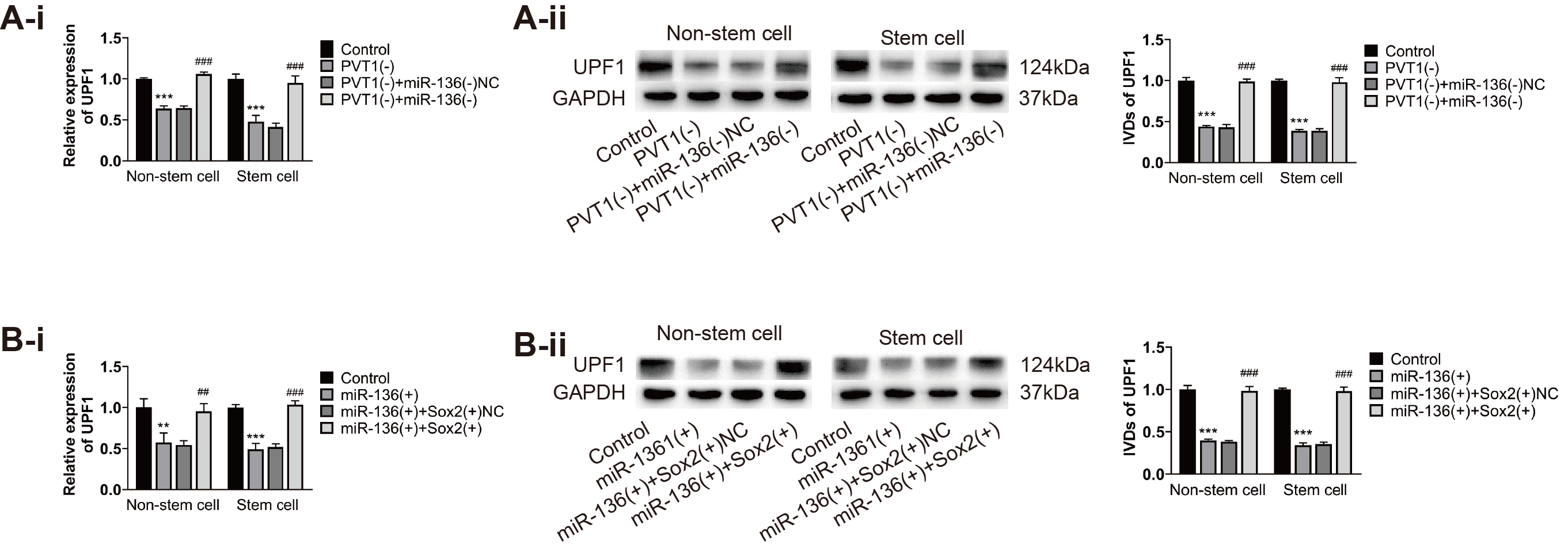

Supplement: Supplementary file 5 — Figure S4 [file 41419_2023_5651_MOESM5_ESM.png]
