# Supplementary material for: PVT1/miR-136/Sox2/UPF1 axis regulates the malignant phenotypes of endometrial cancer stem cells
Source: Cell Death Dis. 2023 Mar 3;14(3):177. doi: 10.1038/s41419-023-05651-0 (PMC9984375; doi:10.1038/s41419-023-05651-0)
Supplement: Supplementary file 6 — Supplementary figure legends [file 41419_2023_5651_MOESM6_ESM.docx]

**Supplementary figure legends**

**Figure S1. A** The CBP resistance concentration curves of endometrial cancer non-stem cells and stem cells. **B-i** The cut-off value of PVT1 was calculated by the ROC curve, and the area under curve (AUC) is 0.7883 (P = 0.0014). **B-ii** Relationship between PVT1 expression and survival in patients with endometrial cancer. **C** The transfection efficiency of PVT1 was detected by qRT-PCR. Data are presented as the means ± SD (n = 3, each group), *P < 0.05, ** P < 0.01, ***P < 0.0001 vs. NC group.

**Figure S2. A-i** The cut-off value of miR-136 was calculated by the ROC curve, and the AUC is 0.7369 (P = 0.0086). **A-ii** Relationship between miR-136 expression and survival in patients with endometrial cancer. **B** The expression of miR-136 and PVT1 in endometrial cancer tissues were negatively correlated (R = -0.3158, P = 0.0188). **C** Changes in the expression of miR-136 were detected by qRT-PCR after PVT1 was regulated. Data are presented as the means ± SD (n = 3, each group), *P < 0.05, ** P < 0.01, ***P < 0.0001 vs. PVT1(+)NC group; #P < 0.05, ##P < 0.01 and ###P < 0.001 vs. PVT1(-)NC group. **D** Changes in the expression of PVT1 were detected by qRT-PCR after miR-136 was regulated. Data are presented as the means ± SD (n = 3, each group), *P < 0.05, ** P < 0.01, ***P < 0.0001 vs. miR-136(+)NC group; #P < 0.05, ##P < 0.01 and ###P < 0.001 vs. miR-136(-)NC group.**E** The transfection efficiency of miR-136 was detected by qRT-PCR. Data are presented as the means ± SD (n = 3, each group), *P < 0.05, ** P < 0.01, ***P < 0.0001 vs. NC group. **Figure S3.** **A** The expression of miR-136 and Sox2 in endometrial cancer tissues were negatively correlated (R = -0.2675, P = 0.0483). **B** Changes in the expression of Sox2 were detected by qRT-PCR and western blot after PVT1 was regulated. Data are presented as the means ± SD (n = 3, each group), *P < 0.05, ** P < 0.01, ***P < 0.0001 vs. PVT1(+)NC group; #P < 0.05, ##P < 0.01 and ###P < 0.001 vs. PVT1(-)NC group. **C** Changes in the expression of Sox2 were detected by qRT-PCR and western blot after miR-136 was regulated. Data are presented as the means ± SD (n = 3, each group), *P < 0.05, ** P < 0.01, ***P < 0.0001 vs. miR-136(+)NC group; #P < 0.05, ##P < 0.01 and ###P < 0.001 vs. miR-136(-)NC group. **D** Changes in the expression of Sox2 were detected by qRT-PCR and western blot after PVT1(-) and miR-136(-) were co-transfected. Data are presented as the means ± SD (n = 3, each group), *P < 0.05, ** P < 0.01, ***P < 0.0001 vs. control group; #P < 0.05, ##P < 0.01 and ###P < 0.001 vs. PVT1(-)+miR-136(-)NC group. **E** The transfection efficiency of Sox2 was detected by qRT-PCR. Data are presented as the means ± SD (n = 3, each group), *P < 0.05, ** P < 0.01, ***P < 0.0001 vs. NC group.

**Figure S4. A** Effects of co-transfected PVT1(-) and miR-136(-) on UPF1 in endometrial cancer non-stem cells and stem cells evaluated by qRT-PCR and western blot. Data are presented as the means ± SD (n = 3, each group), *P < 0.05, ** P < 0.01, ***P < 0.0001 vs. control group; #P < 0.05, ##P < 0.01 and ###P < 0.001 vs. PVT1(-)+miR-136(-)NC group. **B** Effects of co-transfected miR-136 and Sox2 on UPF1 in endometrial cancer non-stem cells and stem cells evaluated by qRT-PCR and western blot. Data are presented as the means ± SD (n = 3, each group), *P < 0.05, ** P < 0.01, ***P < 0.0001 vs. control group; #P < 0.05, ##P < 0.01 and ###P < 0.001 vs. miR-136(+)+Sox2(+)NC group.
