# Supplementary material for: PVT1/miR-136/Sox2/UPF1 axis regulates the malignant phenotypes of endometrial cancer stem cells
Source: Cell Death Dis. 2023 Mar 3;14(3):177. doi: 10.1038/s41419-023-05651-0 (PMC9984375; doi:10.1038/s41419-023-05651-0)
Supplement: Supplementary file 7 — Original Data File [file 41419_2023_5651_MOESM7_ESM.pdf]

Figure 1K-i

Non-stem cell [Control, PVT1(+)NC, PVT1(+)]

CD133 (line 1, lane 1-3)

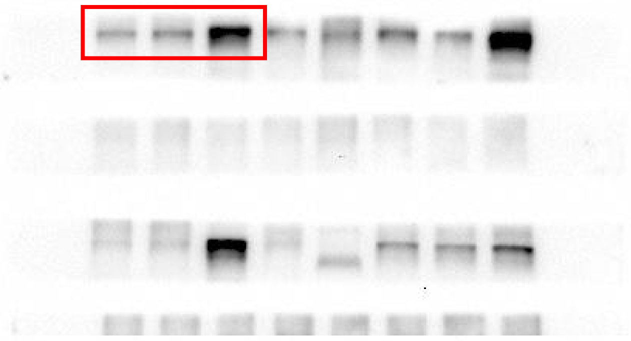

120kDa

CD44 (line 1, lane 1-3)

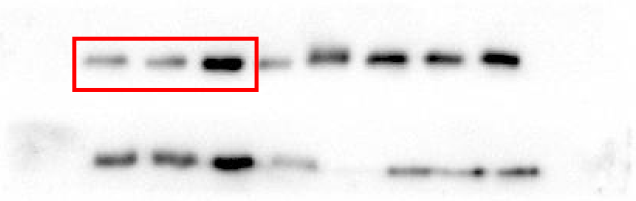

90kDa

Oct4 (line 1, lane 4-6)

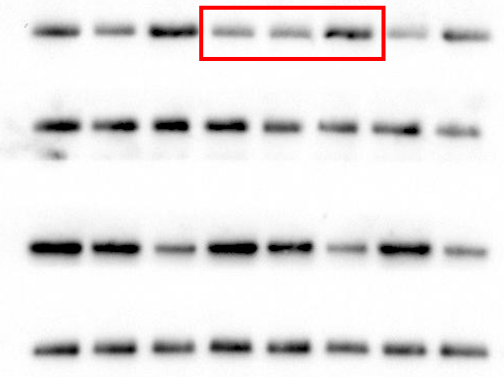

41kDa

Nanog (line 4, lane 1-3)

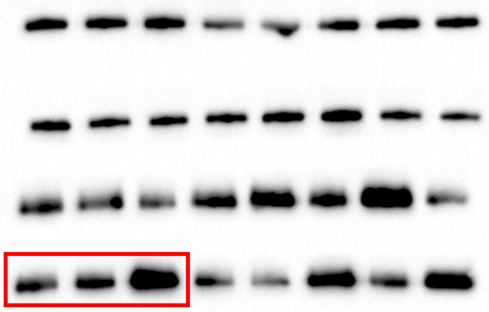

35kDa

GAPDH (line 4, lane 1-3)

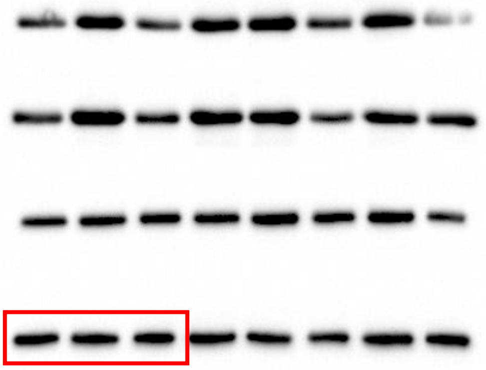

37kDa

Non-stem cell [Control, PVT1(-)NC, PVT1(-)]

CD133 (line 1, lane 1-3)

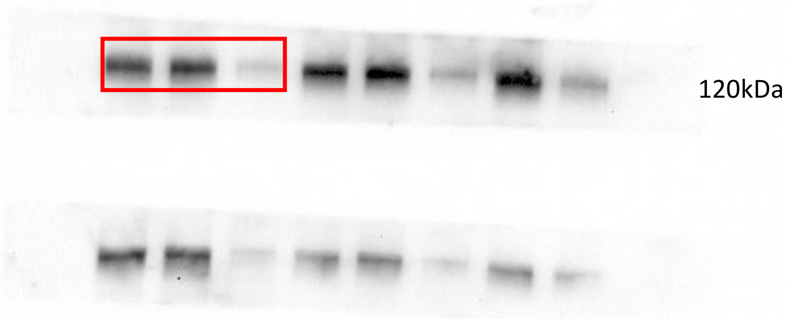

120kDa

CD44 (line 1, lane 1-3)

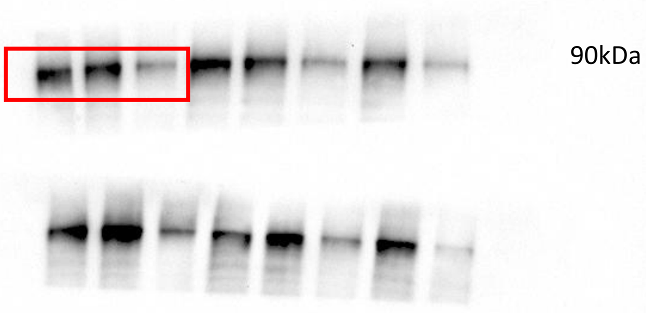

Oct4 (line 2, lane 1-3)

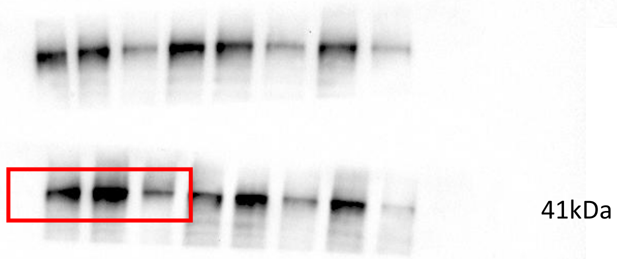

Nanog (line 1, lane 2-4)

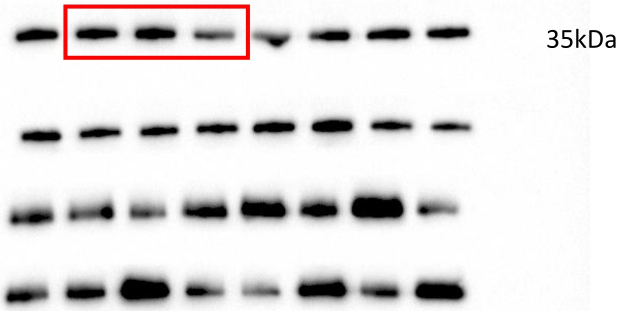

GAPDH (line 3, lane 1-3)

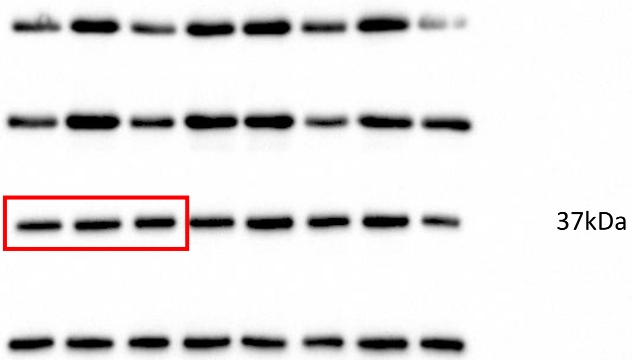

Stem cell [Control, PVT1(+)NC, PVT1(+)]

CD133 (lane 1-3)

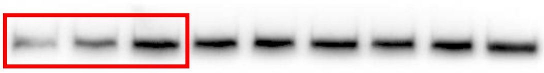

120kDa

CD44 (lane 2-4)

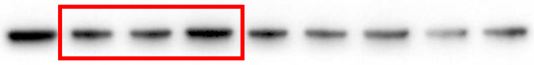

90kDa

Oct4 (line 2, lane 1-3)

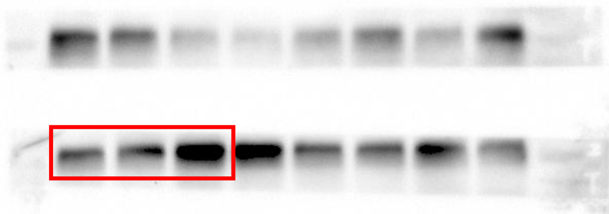

41kDa

Nanog (lane 1-3)

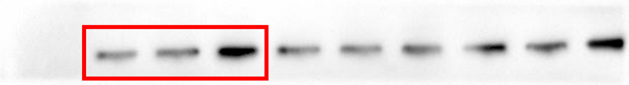

35kDa

GAPDH (line 2, lane 6-8)

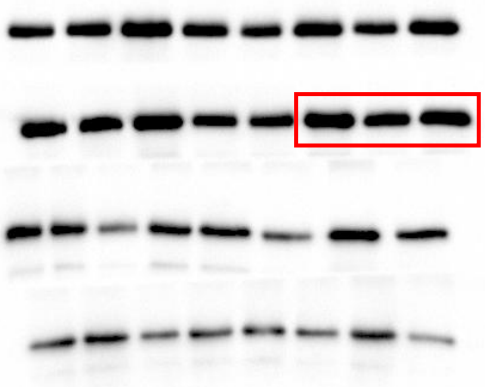

37kDa

Stem cell [Control, PVT1(-)NC, PVT1(-)]

CD133 (lane 1-3)

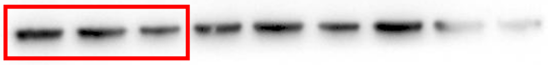

120kDa

CD44 (line 1, lane 3-5)

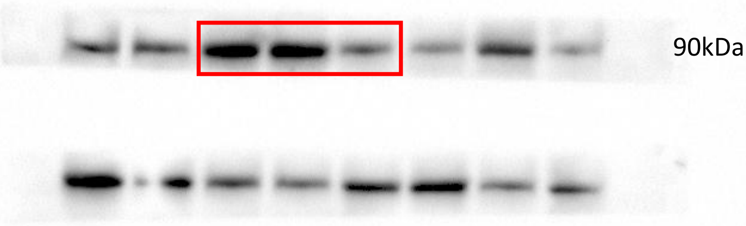

90kDa

Oct4 (line 1, lane 1-3)

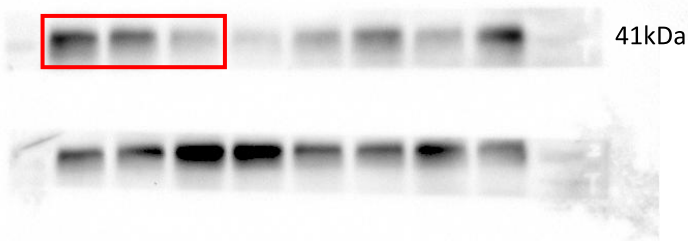

41kDa

Nanog (line 1, lane 4-6)

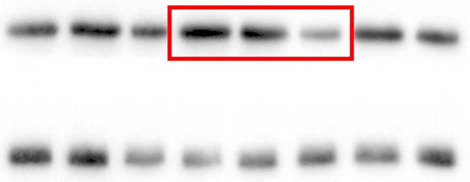

35kDa

GAPDH (line 1, lane 1-3)

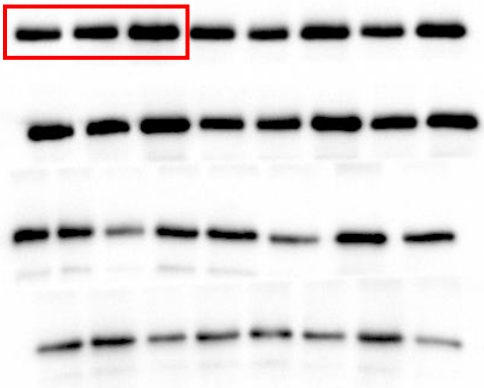

37kDa

Figure 2M-i

Non-stem cell [Control, miR-136(+)NC, miR-136(+)]

CD133 (lane 1-3)

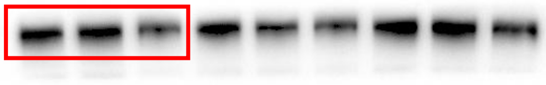

120kDa

CD44 (lane 4-6)

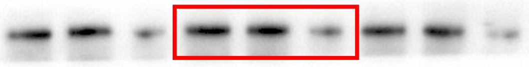

90kDa

Oct4 (lane 1-3)

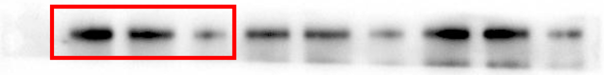

41kDa

Nanog (line 5, lane 1-3)

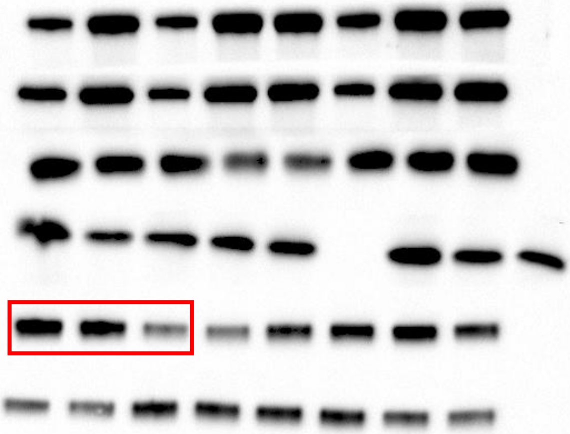

35kDa

GAPDH (line 3, lane 4-6)

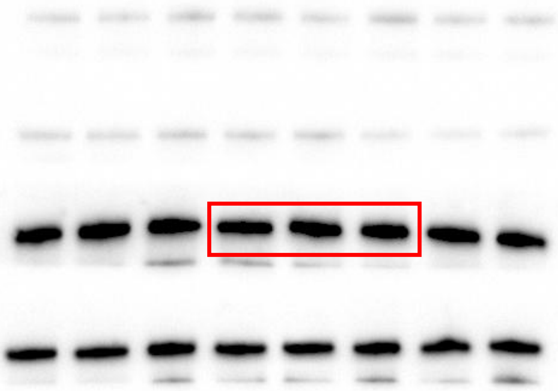

37kDa

Non-stem cell [Control, miR-136(-)NC, miR-136(-)]

CD133 (line 2, lane 1-3)

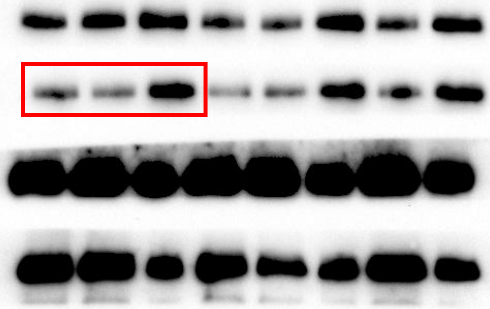

120kDa

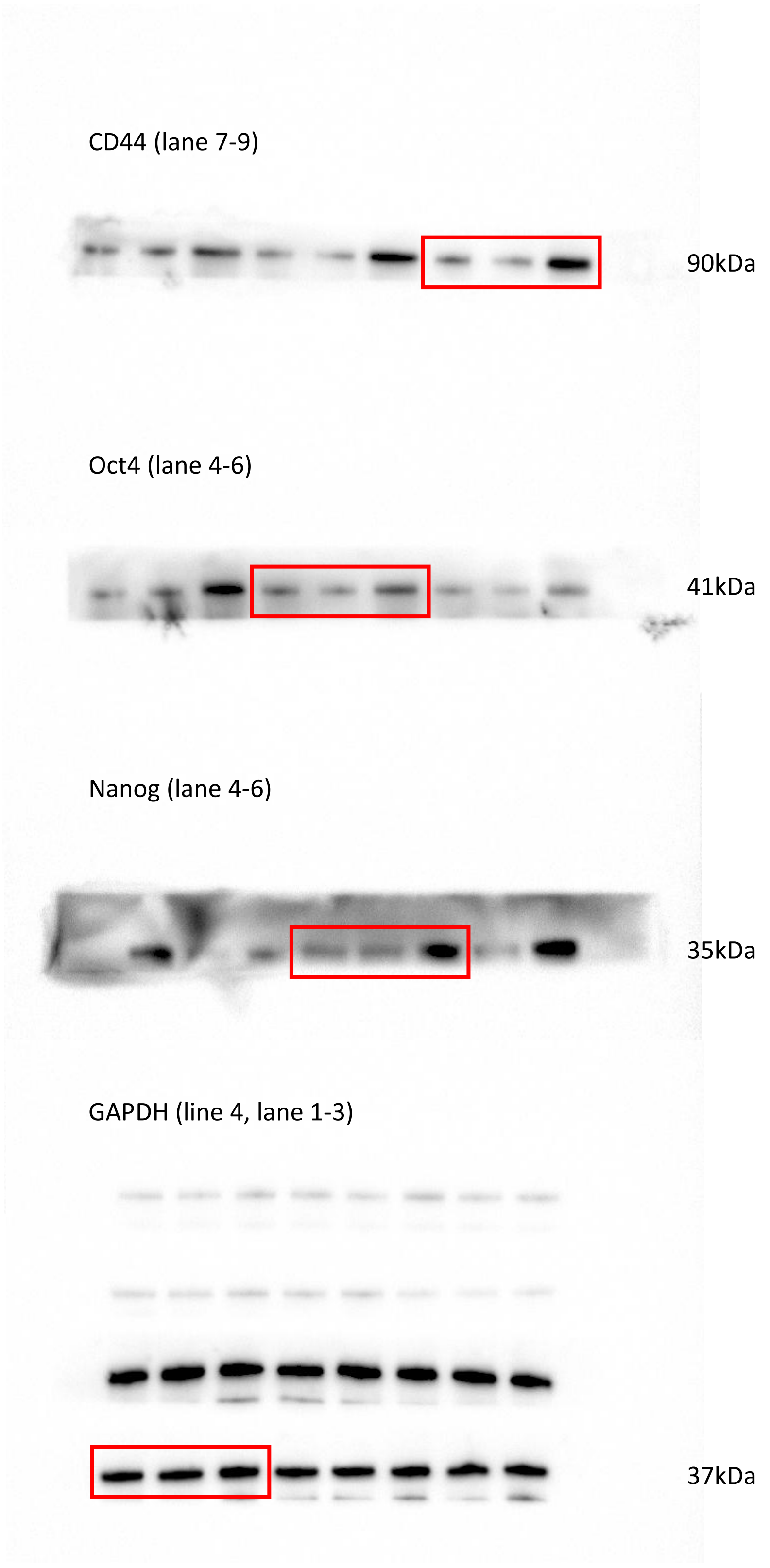
CD44 (lane 7-9)
90kDa
Oct4 (lane 4-6)
41kDa
Nanog (lane 4-6)
35kDa
GAPDH (line 4, lane 1-3)
37kDa

Stem cell [Control, miR-136(+)NC, miR-136(+)]

CD133 (lane 1-3)

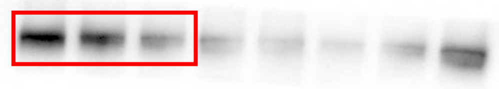

120kDa

CD44 (lane 7-9)

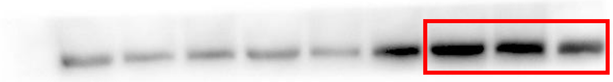

90kDa

Oct4 (lane 4-6)

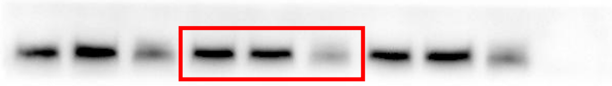

41kDa

Nanog (lane 4-6)

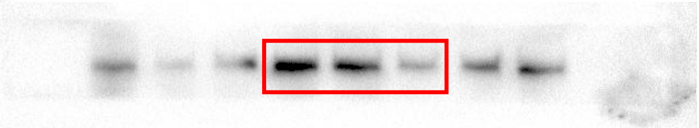

35kDa

GAPDH (lane 7-9)

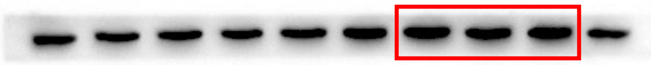

37kDa

Stem cell [Control, miR-136(-)NC, miR-136(-)]

CD133 (line 1, lane 4-6)

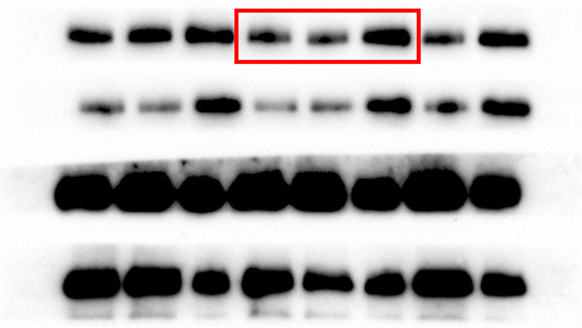

120kDa

CD44 (lane 4-6)

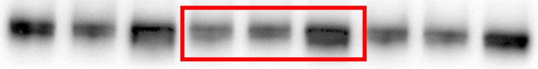

90kDa

Oct4 (lane 1-3)

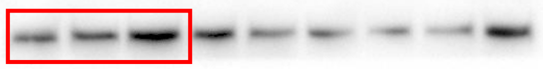

41kDa

Nanog (lane 4-6)

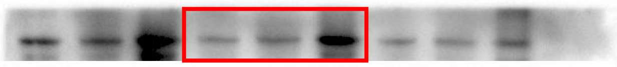

35kDa

GAPDH (lane 2-4)

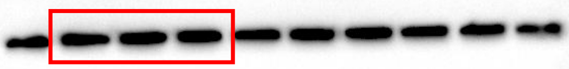

37kDa

Figure 3I-i

Non-stem cell [Control, PVT1(-), PVT1(-)+miR-136(-)NC, PVT1(-)+miR-136(-)]

CD133 (lane 1-4)

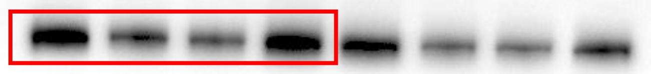

120kDa

CD44 (lane 1-4)

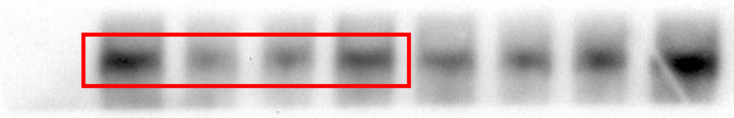

90kDa

Oct4 (lane 5-8)

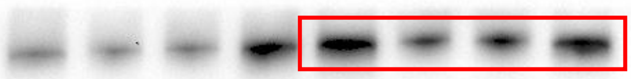

41kDa

Nanog (lane 2-5)

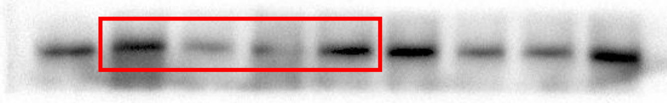

35kDa

GAPDH (lane 2-5)

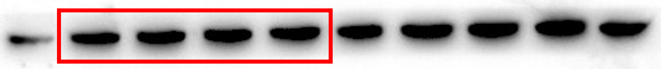

37kDa

Stem cell [Control, PVT1(-), PVT1(-)+miR-136(-)NC, PVT1(-)+miR-136(-)]

CD133 (lane 5-8)

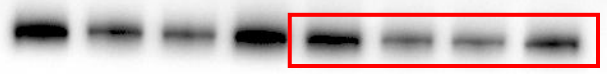

120kDa

CD44 (lane 5-8)

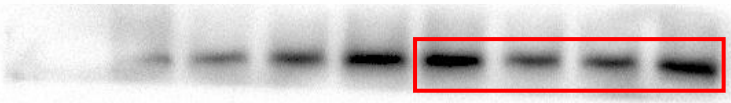

90kDa

Oct4 (lane 5-8)

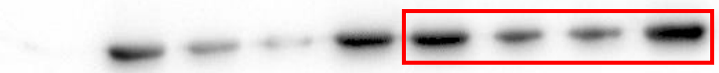

41kDa

Nanog (lane 5-8)

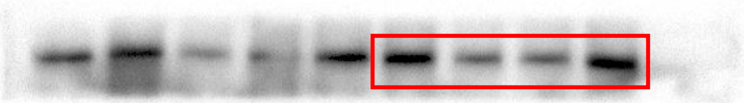

35kDa

GAPDH (lane 6-9)

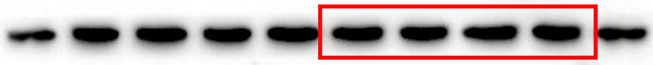

37kDa

Figure 4B

Sox2 (lane 5-8)

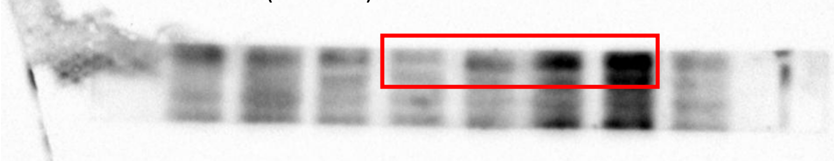

36kDa

β -Actin (lane 5-8)

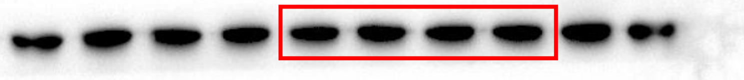

42kDa

Figure 4M-i

Non-stem cell [Control, Sox2(+)NC, Sox2(+)]

CD133 (lane 2-4)

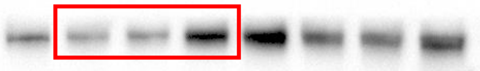

120kDa

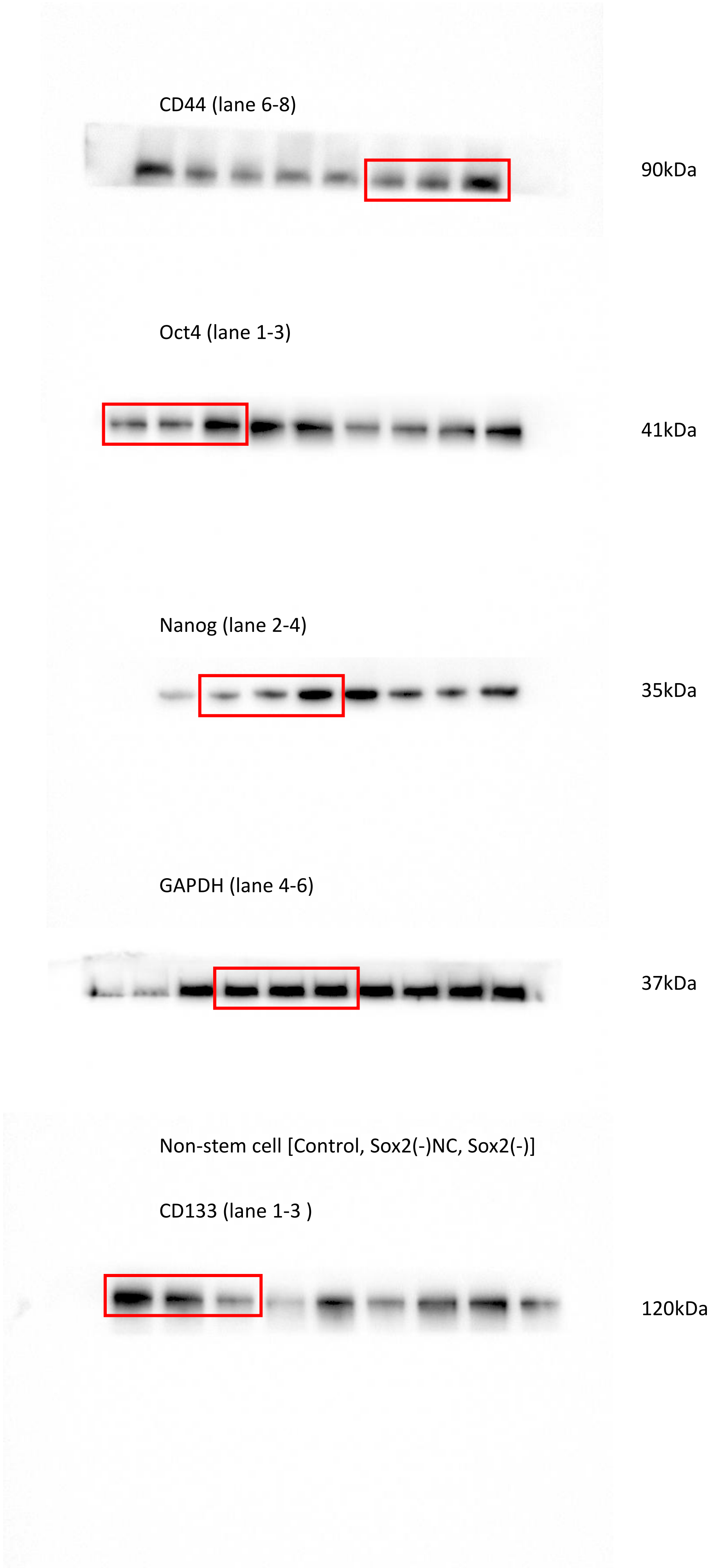
CD44 (lane 6-8)
90kDa
Oct4 (lane 1-3)
41kDa
Nanog (lane 2-4)
35kDa
GAPDH (lane 4-6)
37kDa
Non-stem cell [Control, Sox2(-)NC, Sox2(-)]
CD133 (lane 1-3)
120kDa

CD44 (lane 1-3)

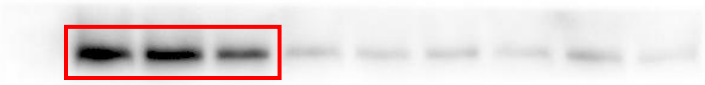

90kDa

Oct4 (lane 1-3)

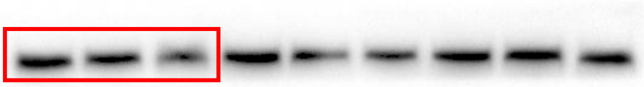

41kDa

Nanog (lane 4-6)

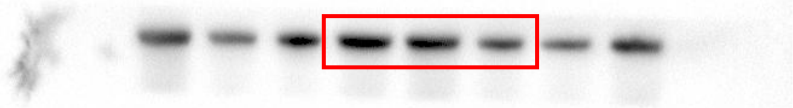

35kDa

GAPDH (lane 4-6)

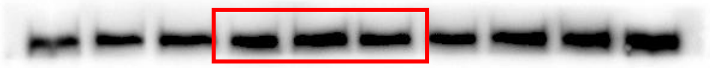

37kDa

Stem cell [Control, Sox2(+)NC, Sox2(+)]

CD133 (lane 5-7)

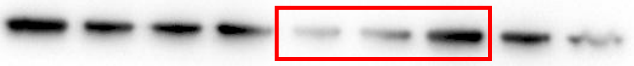

120kDa

CD44 (lane 2-4)

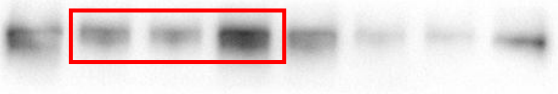

90kDa

Oct4 (lane 2-4)

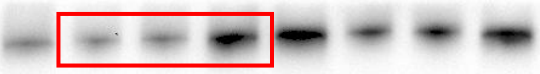

41kDa

Nanog (lane 3-5)

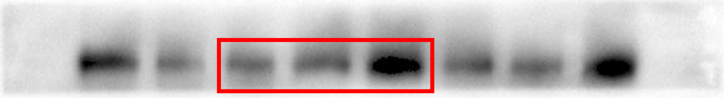

35kDa

GAPDH (lane 2-4)

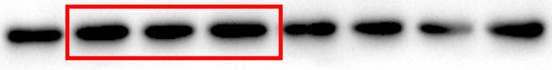

37kDa

Stem cell [Control, Sox2(-)NC, Sox2(-)]

CD133 (lane 1-3)

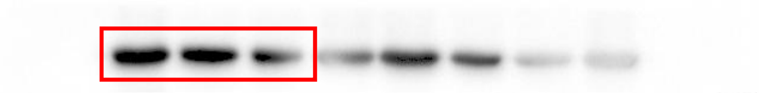

120kDa

CD44 (lane 4-6)

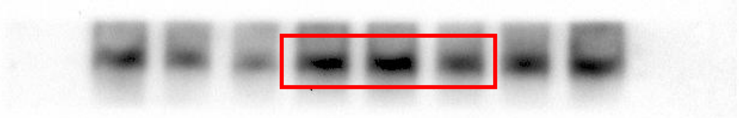

90kDa

Oct4 (lane 4-6)

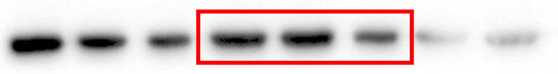

41kDa

Nanog (lane 1-3)

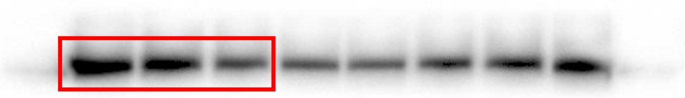

35kDa

GAPDH (lane 2-4)

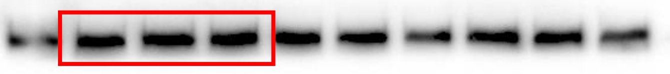

37kDa

Figure 5I-i

Non-stem cell [Control, miR-136(+), miR-136(+)+Sox2(+)NC, miR-136(+)+Sox2(+)]

CD133 (lane 1-4)

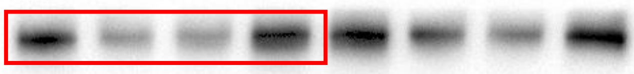

120kDa

CD44 (lane 5-8)

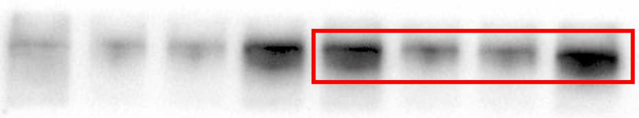

90kDa

Oct4 (lane 1-4)

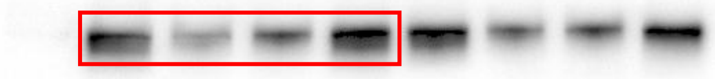

41kDa

Nanog (lane 1-4)

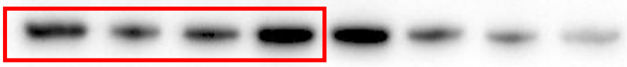

35kDa

GAPDH (lane 2-5)

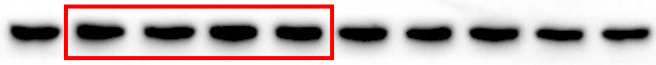

37kDa

Stem cell [Control, miR-136(+), miR-136(+)+Sox2(+)NC, miR-136(+)+Sox2(+)]

CD133 (lane 5-8)

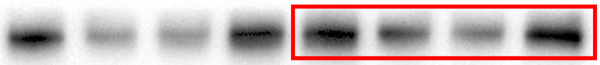

120kDa

CD44 (lane 1-4)

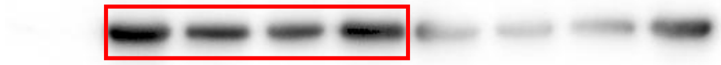

90kDa

Oct4 (lane 5-8)

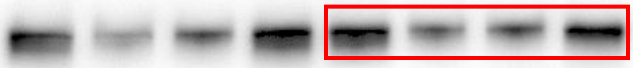

41kDa

Nanog (lane 5-8)

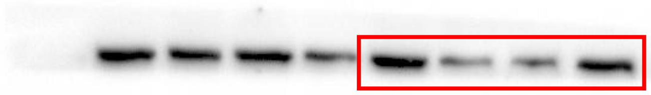

35kDa

GAPDH (lane 2-5)

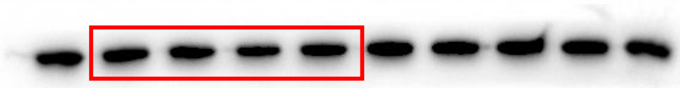

37kDa

Figure 6B

UPF1 (lane 2-5)

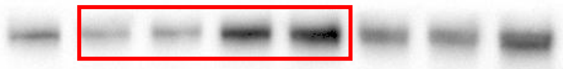

124kDa

β -Actin (lane 3-6)

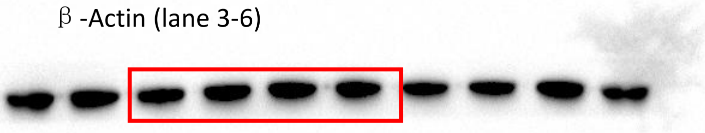

42kDa

Figure 6F-ii

Non-stem cell [Control, PVT1(+)NC, PVT1(+)]

UPF1 (line 3, lane 2-4)

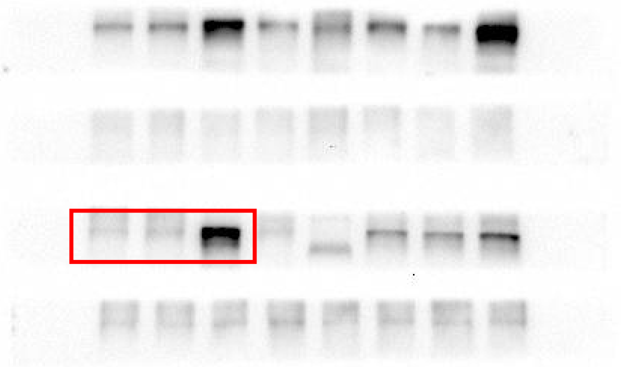

120kDa

GAPDH (line 4, lane 1-3)

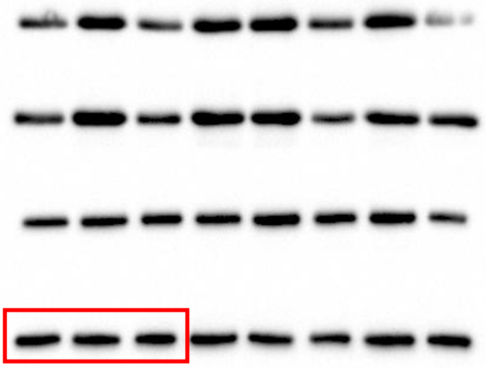

37kDa

Non-stem cell [Control, PVT1(-)NC, PVT1(-)]

UPF1 (line 2, lane 1-3)

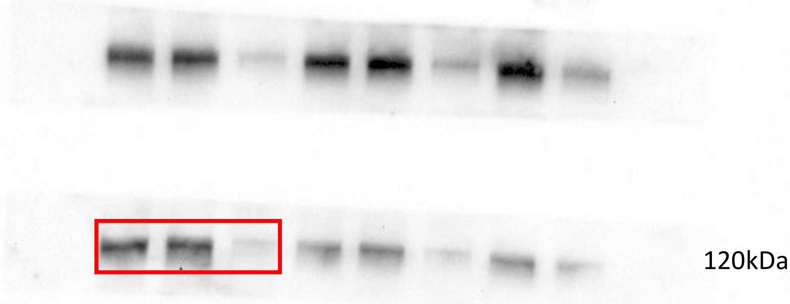

120kDa

GAPDH (line 3, lane 1-3)

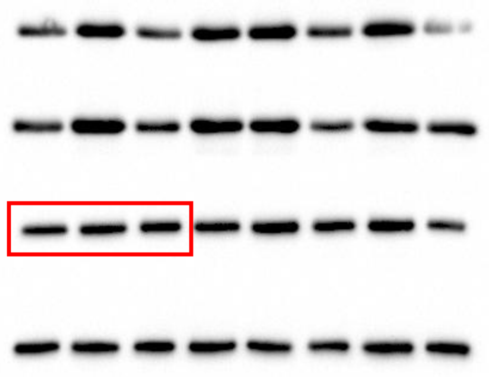

37kDa

Stem cell [Control, PVT1(+)NC, PVT1(+)]

UPF1 (line 2, lane 1-3)

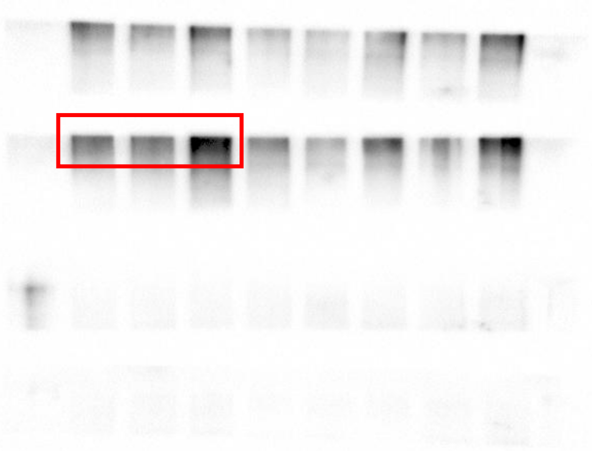

120kDa

GAPDH (line 2, lane 6-8)

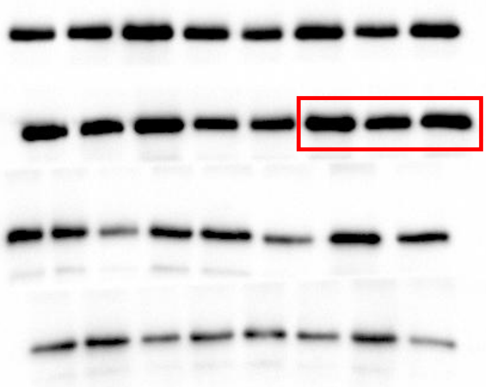

37kDa

Stem cell [Control, PVT1(-)NC, PVT1(-)]

UPF1 (line 1, lane 1-3)

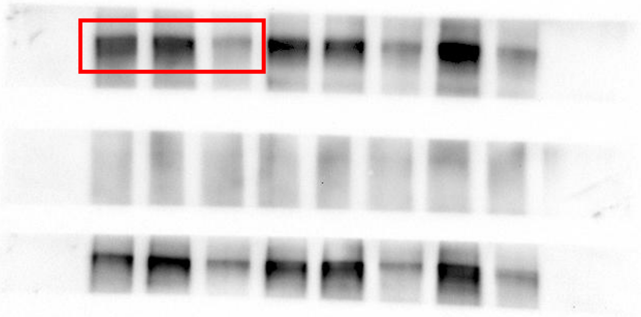

120kDa

GAPDH (line 3, lane 1-3)

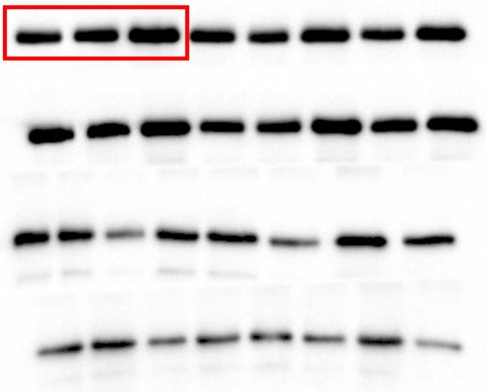

37kDa

Figure 6G-ii

Non-stem cell [Control, miR-136(+)NC, miR-136(+)]

UPF1 (lane 4-6)

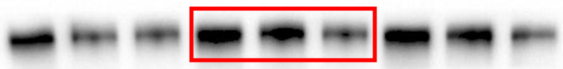

120kDa

GAPDH (line 3, lane 4-6)

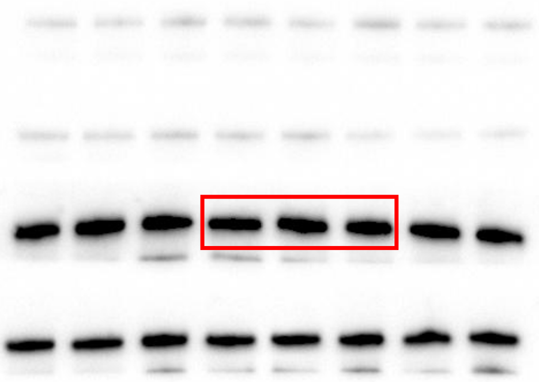

37kDa

Non-stem cell [Control, miR-136(-)NC, miR-136(-)]

UPF1 (lane 4-6)

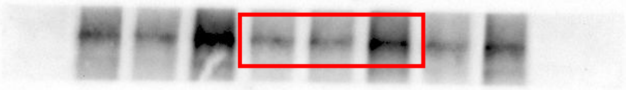

120kDa

GAPDH (line 4, lane 4-6)

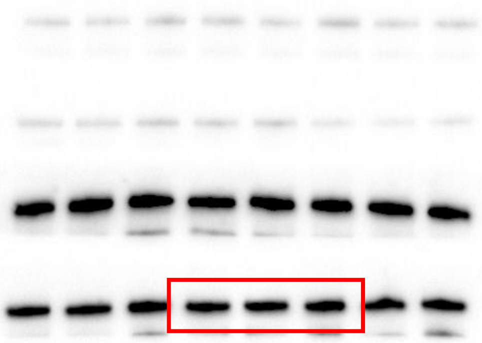

37kDa

Stem cell [Control, miR-136(+)NC, miR-136(+)]

UPF1 (lane 1-3)

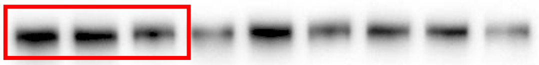

120kDa

GAPDH (lane 7-9)

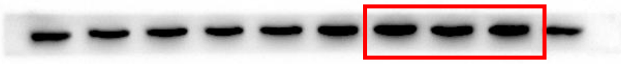

37kDa

Stem cell [Control, miR-136(-)NC, miR-136(-)]

UPF1 (lane 7-9)

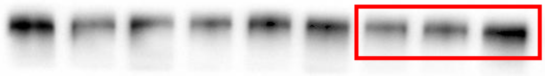

120kDa

GAPDH (lane 2-4)

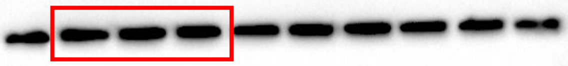

37kDa

Figure 6H-ii

Non-stem cell [Control, Sox2(+)NC, Sox2(+)]

UPF1 (lane 1-3)

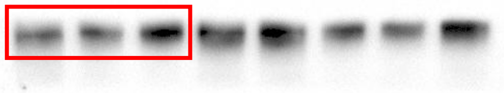

120kDa

GAPDH (lane 4-6)

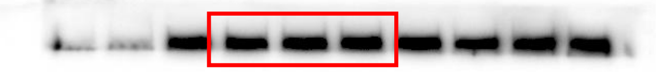

37kDa

Non-stem cell [Control, Sox2(-)NC, Sox2(-)]

UPF1 (lane 4-6)

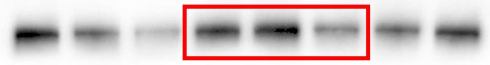

120kDa

GAPDH (lane 4-6)

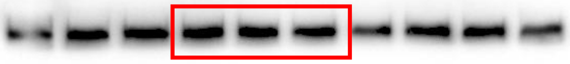

37kDa

Stem cell [Control, Sox2(+)NC, Sox2(+)]

UPF1 (lane 2-4)

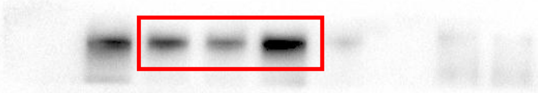

120kDa

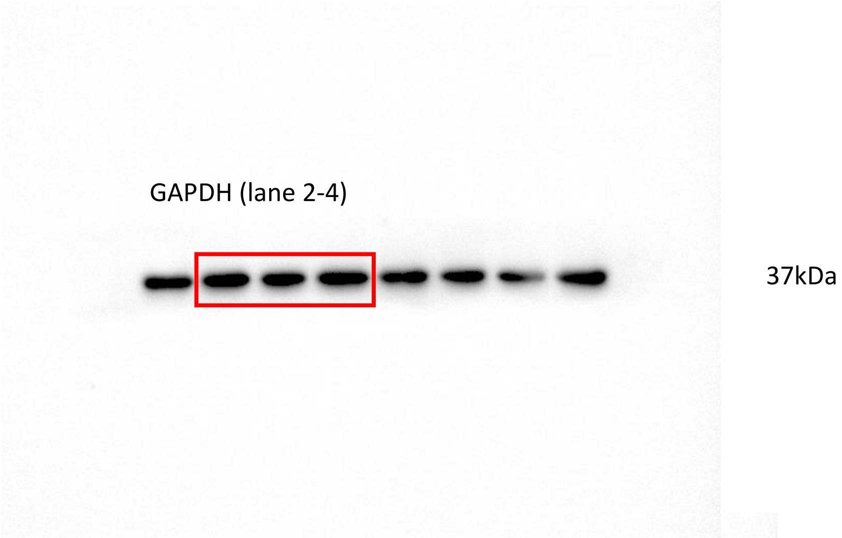
GAPDH (lane 2-4)
37kDa

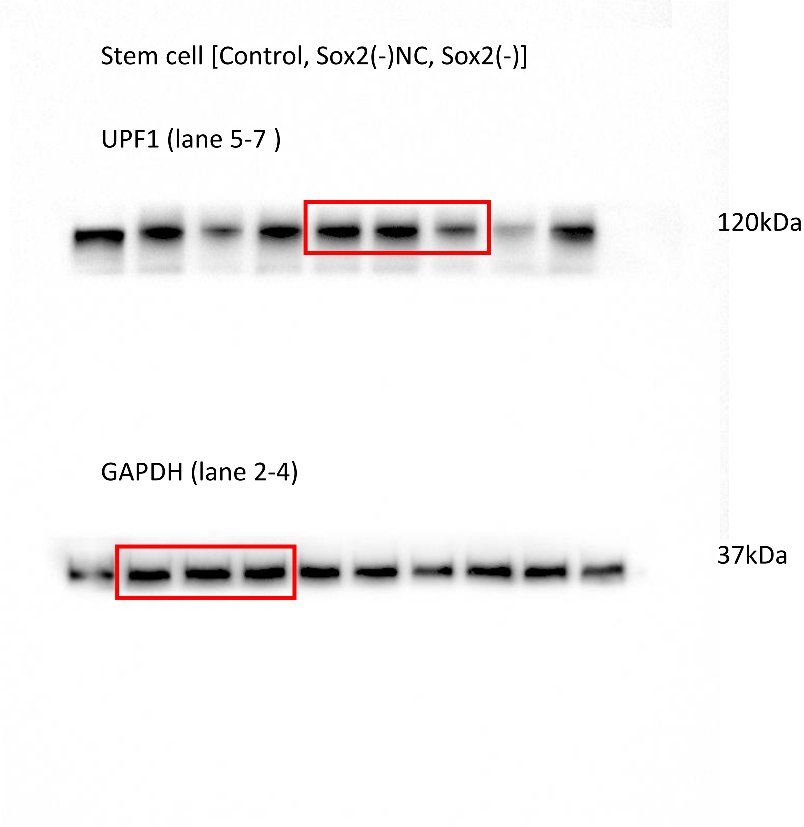
Stem cell [Control, Sox2(-)NC, Sox2(-)]
UPF1 (lane 5-7)
120kDa
GAPDH (lane 2-4)
37kDa

Figure 7C-i

Non-stem cell [Control, PVT1(-)NC, PVT1(-), miR-136(+)NC, miR-136(+), PVT1(-)+miR-136(+)]

UPF1 (lane 2-7)

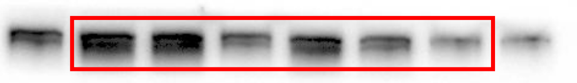

124kDa

CD133 (lane 2-7)

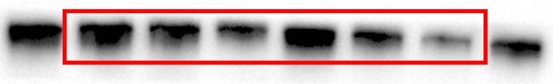

120kDa

CD44 (lane 2-7)

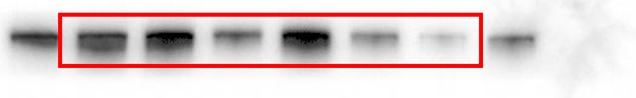

90kDa

Oct4 (lane 2-7)

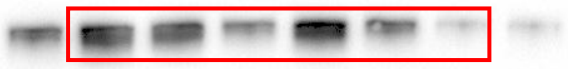

41kDa

Sox2 (lane 1-6)

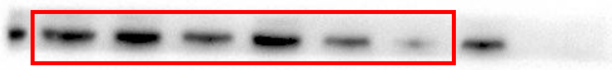

36kDa

Nanog (lane2-7)

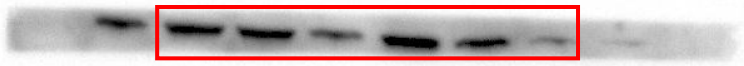

35kDa

GAPDH (lane 2-7)

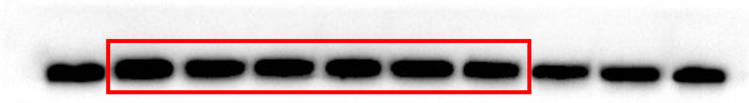

37kDa

Stem cell [Control, PVT1(-)NC, PVT1(-), miR-136(+)NC, miR-136(+), PVT1(-)+miR-136(+)]

UPF1 (lane 2-7)

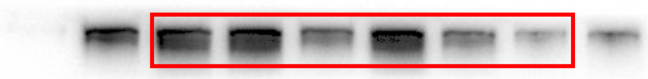

124kDa

CD133 (lane 2-7)

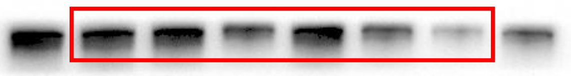

120kDa

CD44 (lane 2-7)

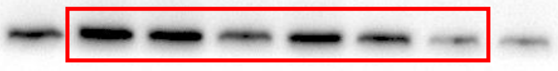

90kDa

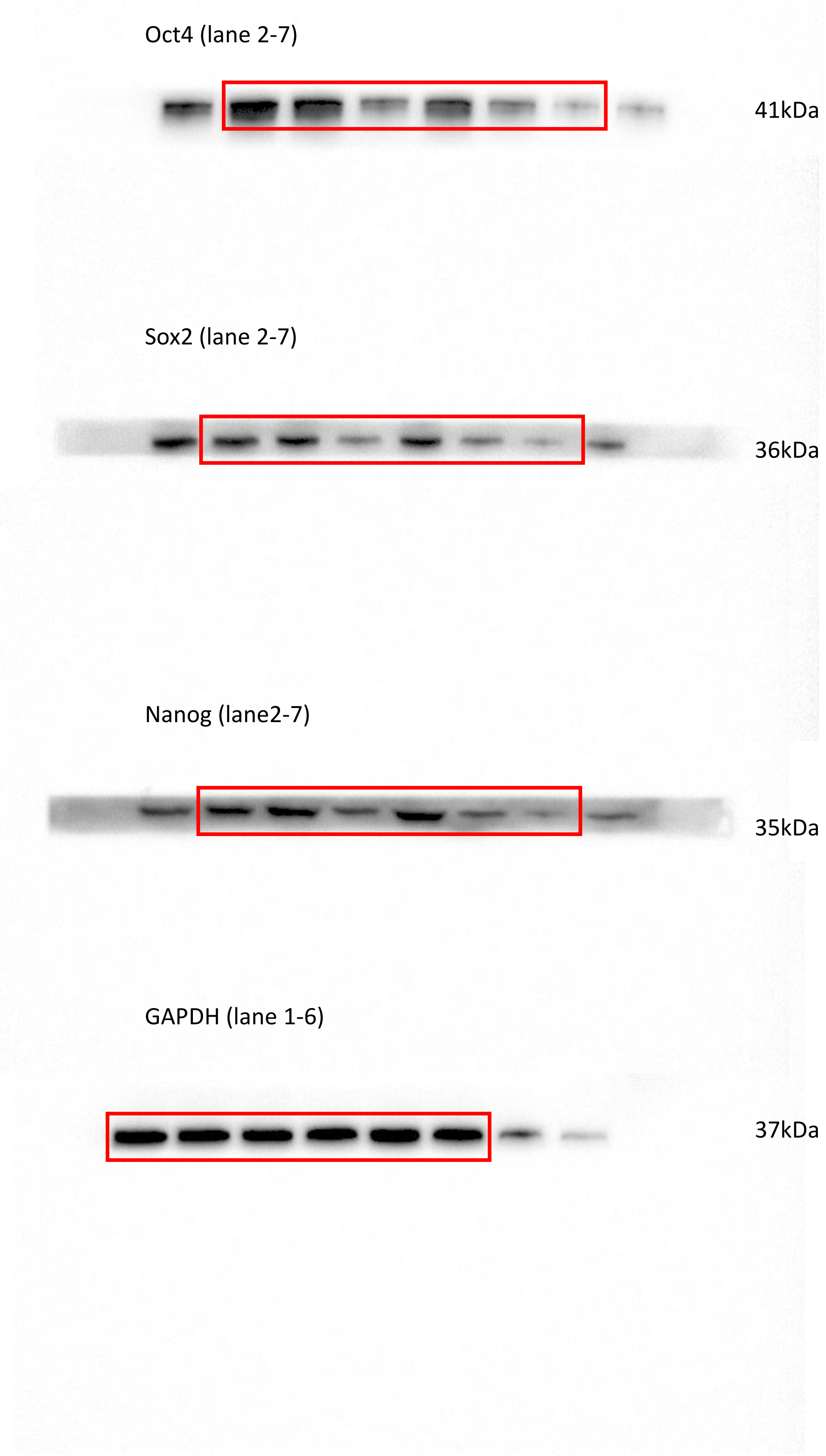

Oct4 (lane 2-7)
41kDa
Sox2 (lane 2-7)
36kDa
Nanog (lane2-7)
35kDa
GAPDH (lane 1-6)
37kDa

Figure S3B-ii

Non-stem cell [Control, PVT1(+)NC, PVT1(+)]

Sox2 (line 6, lane 1-3)

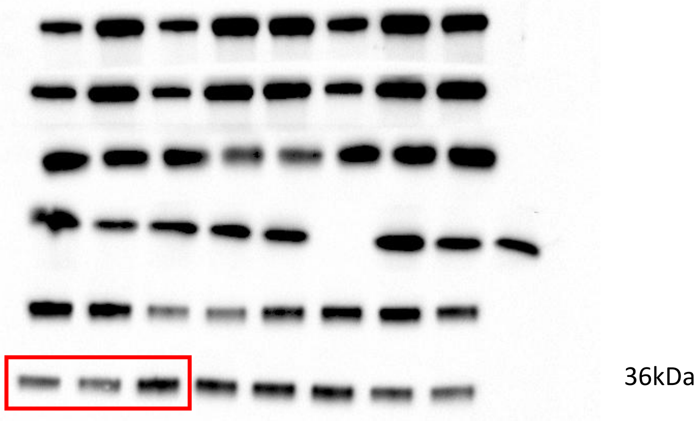

GAPDH (line 4, lane 1-3)

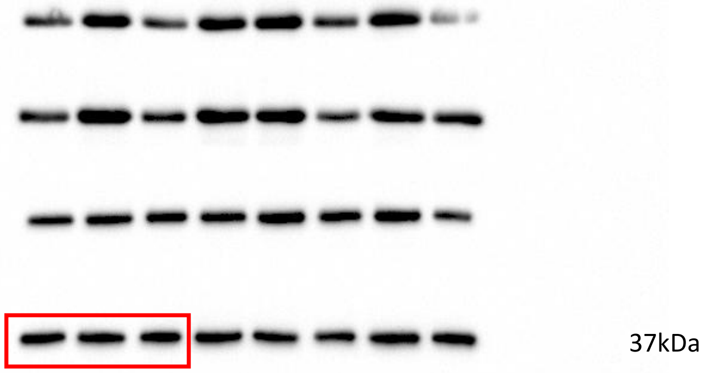

Non-stem cell [Control, PVT1(-)NC, PVT1(-)]

Sox2 (line 3, lane 1-3)

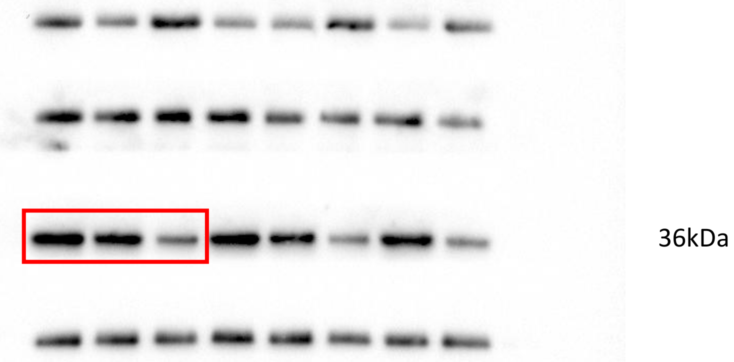

GAPDH (line 4, lane 1-3)

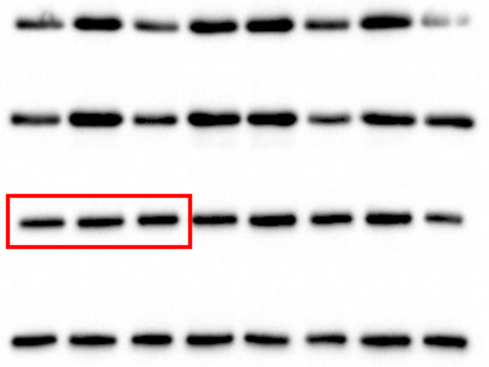

37kDa

Stem cell [Control, PVT1(+)NC, PVT1(+)]

Sox2 (line 2, lane 3-5)

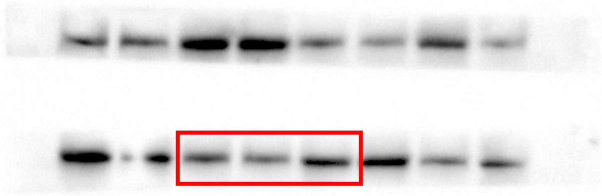

36kDa

GAPDH (line 2, lane 6-8)

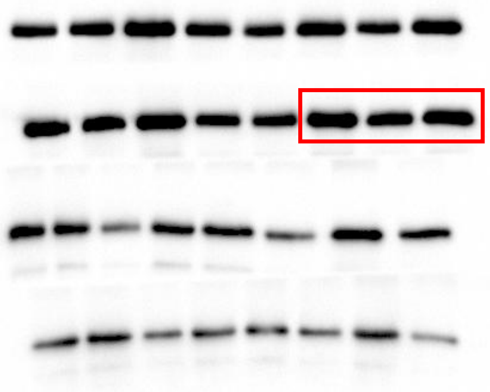

37kDa

Stem cell [Control, PVT1(-)NC, PVT1(-)]

Sox2 (lane 2-4)

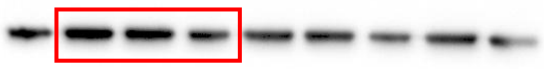

36kDa

GAPDH (line 1, lane 1-3)

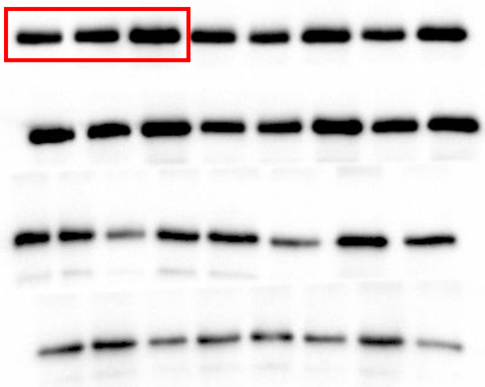

37kDa

Figure S3C-ii

Non-stem cell [Control, miR-136(+)NC, miR-136(+)]

Sox2 (line 1, lane 1-3)

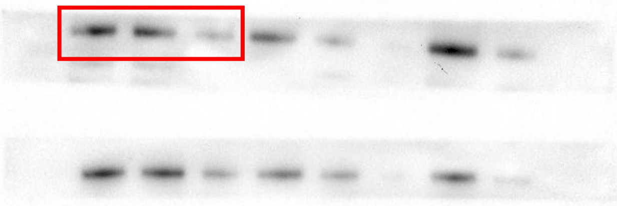

36kDa

GAPDH (line 3, lane 4-6)

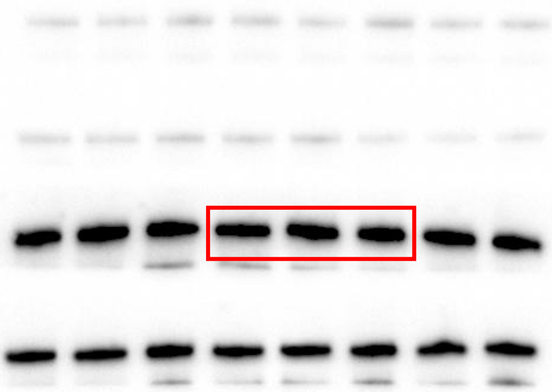

37kDa

Non-stem cell [Control, miR-136(-)NC, miR-136(-)]

Sox2 (lane 6-8)

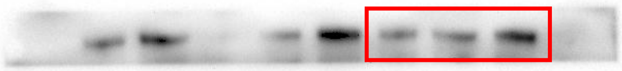

36kDa

GAPDH (line 4, lane 1-3)

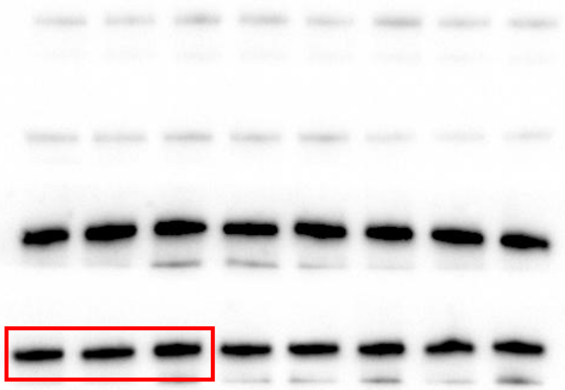

37kDa

Stem cell [Control, miR-136(+)NC, miR-136(+)]

Sox2 (line 2, lane 1-3)

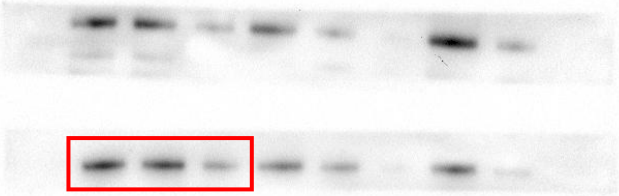

36kDa

GAPDH (lane 7-9)

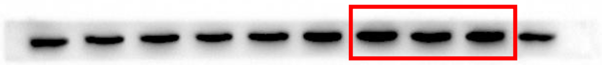

37kDa

Stem cell [Control, miR-136(-)NC, miR-136(-)]

Sox2 (lane 5-7)

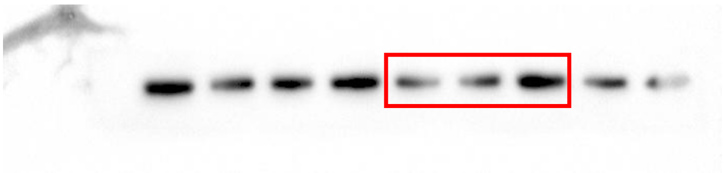

36kDa

GAPDH (lane 2-4)

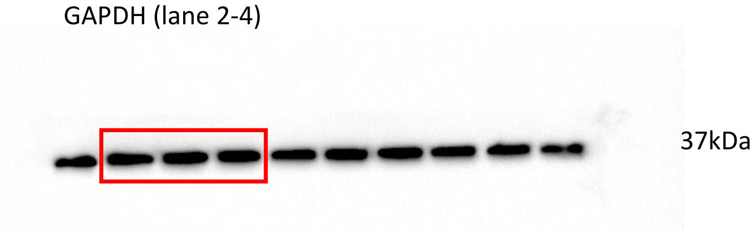

37kDa

Figure S3D-ii

Non-stem cell [Control, PVT1(-), PVT1(-)+miR-136(-)NC, PVT1(-)+miR-136(-)]

Sox2 (lane 5-8)

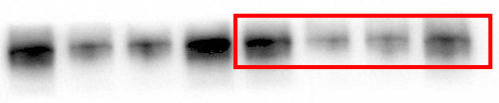

36kDa

GAPDH (lane 2-5)

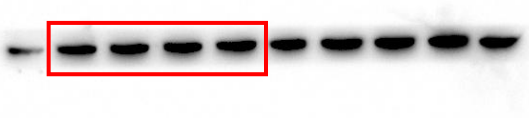

37kDa

Stem cell [Control, PVT1(-), PVT1(-)+miR-136(-)NC, PVT1(-)+miR-136(-)]

Sox2 (lane 5-8)

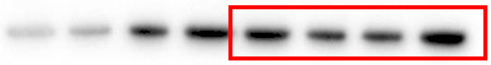

36kDa

GAPDH (lane 6-9)

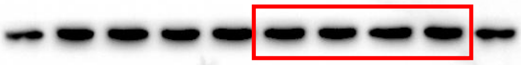

37kDa

Figure S4B

Non-stem cell [Control, PVT1(-), PVT1(-)+miR-136(-)NC, PVT1(-)+miR-136(-)]

UPF1 (lane 1-4)

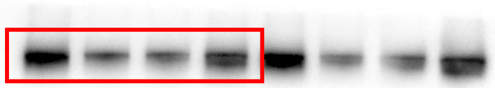

124kDa

GAPDH (lane 2-5)

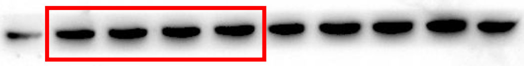

37kDa

Stem cell [Control, PVT1(-), PVT1(-)+miR-136(-)NC, PVT1(-)+miR-136(-)]

UPF1 (lane 5-8)

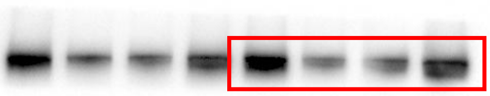

124kDa

GAPDH (lane 2-5)

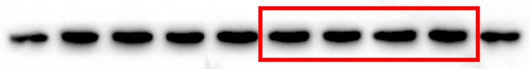

37kDa

Figure S4D

Non-stem cell [Control, miR-136(+), miR-136(+)+Sox2(+)NC, miR-136(+)+Sox2(+)]

UPF1 (lane 1-4)

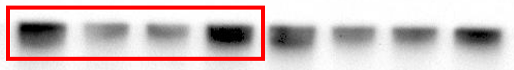

124kDa

GAPDH (lane 2-5)

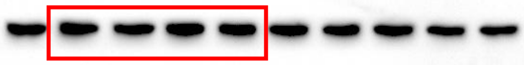

37kDa

Stem cell [Control, miR-136(+), miR-136(+)+Sox2(+)NC, miR-136(+)+Sox2(+)]

UPF1 (lane 5-8)

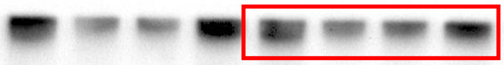

124kDa

GAPDH (lane 2-5)

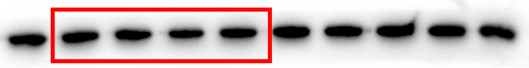

37kDa
